# HMQ-ES-Stack-GBR: A Hybrid Ensemble Learning Model for Mechanical and Physical Quality Prediction in FDM 3D Printing

**DOI:** 10.3390/mi17070859

**Published:** 2026-07-18

**Authors:** Elif Aktepe, Uçman Ergün

**Affiliations:** 1 Afyon Vocational School, Electronics and Automation Department, Afyon Kocatepe University, Afyonkarahisar 03200, Turkey; 2 Engineering Faculty, Biomedical Engineering Department, Afyon Kocatepe University, Afyonkarahisar 03200, Turkey; uergun@aku.edu.tr

**Keywords:** machine learning, 3D printing, optimization, hybrid ensemble learning, quality prediction

## Abstract

In Fusion Deposition Modeling-based manufacturing, process parameters affect the mechanical and physical properties of the print. Considering these properties, accurately predicting print quality is essential. This is where machine learning (ML) models for three-dimensional (3D) print quality prediction come to the forefront. In this study, a dataset was prepared under strict operational measurement standards—utilizing the Interquartile Range (IQR) method for data sanitization—encompassing 10 material types, 2 printer types, and 4 printing parameters. Five hundred different sample combinations were prepared and printed in sets of three according to ISO 527-2 Type 4 standard dimensions. Tensile, hardness, and surface roughness tests were applied to the prepared samples. Using this validated dataset, a Hybrid Multi-Material Quality–Ensemble System–Stacking–Gradient Boosting Regressor (HMQ-ES-Stack-GBR) architecture is proposed as a diagnostic framework for multi-output quality prediction. Particularly in terms of quality outputs such as tensile strength, hardness, and surface roughness, while also providing a quantitative analysis of the effect of material type on print quality. Furthermore, a multi-objective optimization pipeline integrating three distinct meta-heuristic algorithms—Non-dominated Sorting Genetic Algorithm II (NSGA-II), Particle Swarm Optimization (PSO), and Grey Wolf Optimizer (GWO)—was coupled with the framework to systematically derive material-specific optimal processing parameter configurations. Furthermore, the study shows that open-system printers exhibit higher prediction errors than closed-system printers. Reflecting system-induced variability rather than full hardware independence. Although the study is limited to internal validation within the current experimental design and includes material imbalance across filament groups, the findings suggest that the proposed framework provides a promising diagnostic decision-support tool for pre-print quality estimation within the studied dataset. By accurately reflecting rather than physically overcoming manufacturing variability, it supports decision-making processes through pre-print quality estimation, thereby enabling proactive interventions that reduce raw material, time, and energy losses.

## 1. Introduction

As a result of digitalization in the production cycle, the demand for additive manufacturing (AM) technologies is rapidly increasing. The basis of AM processes rests on three-dimensional (3D) printing technologies [1], most commonly based on Fusion Deposition Modeling (FDM) [2]. Although FDM-based technologies are preferred for their cost-effectiveness, wide range of materials, and ease of use, variations in print quality can occur due to thermal, electrical, or mechanical factors during the printing process [3]. This variability may stem from the multivariate, complex, and interactive relationships among different printing parameters. These include parameters such as layer thickness [4], infill pattern and ratio [5], print speed [6], temperature conditions [7], and dimensional accuracy [8]. Traditional optimization approaches currently in use are often insufficient for explaining the complex relationships among these pressure parameters. This is precisely where machine learning (ML) and deep learning (DL) based models, which have recently gained popularity in many fields, come to the forefront in FDM processes. Due to the high success rates of these models in FDM processes, there are many studies in the current literature [9,10,11,12,13,14]. In this context, the current literature on 3D printing quality assessment includes numerous studies focusing on predicting quality outcomes based on printing, such as tensile strength [15,16,17,18,19,20], hardness [21,22,23,24], mechanical properties, and surface roughness [25,26,27,28,29] for physical properties.

However, most of these studies have focused on a single quality assessment metric, a limited material group, a limited number of samples, or traditional ML algorithms [30,31,32,33]. This clearly highlights the need for next-generation hybrid prediction architectures that are adaptable to different quality outputs and integrate multiple models. The proposed HMQ-ES-Stack-GBR framework is conceptually grounded in ensemble learning and stacked generalization theory. Ensemble learning combines multiple predictive models to improve robustness and predictive accuracy, whereas stacking employs a meta-learner to integrate the outputs of several base learners [34,35]. Foundational studies demonstrated that stacked architectures can outperform individual learners by exploiting complementary predictive behaviors. In addition, gradient boosting approaches have proven highly effective for modeling nonlinear relationships in complex datasets [36]. Building upon these established principles, the HMQ-ES-Stack-GBR framework integrates XGBoost, Random Forest, and Gradient Boosting Regressor within a hierarchical stacking architecture specifically designed for multi-material and multi-output quality prediction in FDM-based 3D printing environments.

In the current literature, Mulugundam et al., in their ML-based studies on predicting quality outputs in FDM-based 3D printing processes, investigated the effects of material density and tensile strength across combinations of layer height, infill percentage, and printing speed parameters [37]. Using different ML prediction models, they presented that the Extreme Gradient Boosting (XGB) regressor has the lowest error statistics and is the most successful model for parameter optimization [37]. Ogunsanya et al. studied backpropagation, radial basis, forward, and generalized regression neural networks used to predict dimensional accuracy, porosity, and tensile strength [38]. Nguyen et al. used response surface methodology and Artificial Neural Networks (ANNs) for pattern recognition to predict the tensile strength of Acrylonitrile Butadiene Styrene (ABS) material using five basic printing parameters, including extrusion temperature, layer thickness, printing speed, number of strokes, and infill density, followed by the application of a genetic algorithm and a multi-criteria decision-making algorithm for optimization [39]. These hybrid approaches demonstrate a successful correlation between tensile strength and production time [39]. Panico et al. predicted the tensile strength, strain at maximum strain, and elastic modulus of ABS samples depending on the parameters of layer thickness, extrusion temperature, printing speed, and infill pattern [40]. They noted that integrating RF models into the NSGA-II algorithm provides effective multi-objective optimization [40]. Özkül et al. reported R^2^ ≈ 0.99 for estimating hardness and surface roughness with the KSTAR algorithm and tensile and flexural strength with the MLP algorithm using only 27 ABS samples [41]. Nagarjun et al. demonstrated that Gaussian Process Regression accurately predicted the tensile strength of Polylactic Acid (PLA) material with an accuracy of 88.34% [42]. Saylık et al. used the Support Vector Machine algorithm to predict the tensile strength of 48 PLA samples, achieving an R^2^ of 0.87 [43].

In studies focusing on surface roughness prediction, various machine learning approaches have demonstrated high predictive performance. Soundararajan et al. reported that RF and J48 models achieved R^2^ values of approximately 0.93–0.95 and identified printing speed and layer height as the most influential parameters [44]. Similarly, hybrid optimization and ANN-based approaches have been successfully applied to roughness reduction and process optimization in PLA materials [45]. Tzotzis et al. reported an R^2^ value of 0.99 for surface roughness prediction using a dataset of fiber-reinforced PETG specimens [46]. Islahudin et al. demonstrated that XGBoost achieved an R^2^ value of 0.97 in surface roughness estimation [47]. Similarly, AdaBoost-based approaches have been reported to achieve R^2^ values approaching 0.99 for roughness prediction tasks [48]. In a manufacturing optimization study, machine learning-assisted regression models reduced the prediction error of surface quality to 3.17% [49]. Furthermore, XGBoost achieved prediction performances close to R^2^ = 0.99 in biomedical precision tooth prototyping applications [50].

Similarly, studies involving different material classes have shown that machine learning models can effectively predict mechanical and physical properties across a wide range of composite and engineering materials. Ensemble learning approaches have been applied to PLA/HAp composites [51], PLA-CF materials [52], nylon–aramid composites [53], sea coral sand–clay mixtures [54], and walnut-shell-reinforced PLA structures [55]. In addition, recent review studies have highlighted the growing role of machine learning in biomedical additive manufacturing applications, particularly in the design and optimization of bone implants [56].

Furthermore, recent developments in additive manufacturing have extended beyond quality prediction toward Digital Twin-assisted optimization [57], inverse design methodologies [58], Internet of Things (IoT)-integrated manufacturing systems [59], accelerated alloy development [60], and active learning-based material property prediction [61]. These studies collectively demonstrate the expanding role of data-driven methodologies in modern additive manufacturing environments. There are also studies on error and defect detection in 3D printing based on DL [62].

Furthermore, recent breakthrough studies in metal additive manufacturing have successfully integrated physics-based constraints with advanced Explainable AI (XAI) frameworks—such as SHapley Additive exPlanations (SHAP) and counterfactual explanations (CE)—to dynamically evaluate and mitigate in-process anomalies like overheating in laser powder bed fusion (L-PBF) systems by prescribing actionable, domain-specific parameter modifications [63]. Similarly, recent studies in the literature have utilized Bayesian-optimized ML models to predict complex mechanical properties, such as the Charpy impact strength of high-performance short carbon fiber-reinforced thermoplastic composites (PAHT-CF) fabricated via FDM, successfully explaining the nonlinear synergistic interactions of dominant manufacturing parameters like printing orientation through SHAP analysis [64]. In a similar vein, recent state-of-the-art frameworks have combined high-fidelity XGBoost regressors with SHAP to map the multi-response performance (tensile strength, surface roughness, and elongation) of 3D printed parts across an extensive array of parameters—such as layer height, nozzle temperature, and infill density—revealing that localized interactions, like the positive correlation between layer height and roughness, can be successfully untangled via interpretable ML [65].

When all these studies in the current literature are evaluated together, they show that the quality prediction performance in FDM-based 3D printing processes is sensitive not only to the algorithm used but also to the type of filament chosen and the targeted quality output. Studies across different filaments show that models excel at different quality metrics, suggesting that a single predictive model may face challenges in offering a universal solution for all quality outputs. However, the fact that the majority of these studies have been conducted with small datasets highlights the need for larger-scale experimental frameworks to evaluate model performance across heterogeneous materials. This situation indicates that fixed-weight ensemble approaches and singular models may not fully capture the industrial complexity of multidimensional, heterogeneous production data within certain constraints. In the current literature, piecewise and specific prediction models for different materials and quality outputs are predominantly discussed.

The primary motivation for this study is to address a methodological gap identified in previous studies, where approaches often focus on a single polymer group, are trained on limited sample sizes, and show limited capacity in modeling diverse material behaviors across heterogeneous datasets. A significant portion of the studies in the literature use limited datasets, which can limit the generalizability of ML models used in quality prediction and their prediction performance on different filament types. In this study, a sample range of 500 samples was created using 10 different materials, ranging from elastomeric materials such as thermoplastic polyurethane (TPU) to carbon fiber-reinforced PLA composites and functional filaments such as recycled polyethylene terephthalate (rPET), phosphorus-doped PLA, and PETG. In this dataset, 500 combinations were obtained with four printing parameters and three levels of each parameter. A total of 1500 sample prints were made by printing 3 replicate samples from each combination. Tensile, hardness, and roughness tests were applied to the prepared samples.

Another important aspect of the study is improving the industrial relevance of the model by simultaneously using open and closed case 3D printer systems and by examining the effects of thermal environmental conditions on the production processes, thereby capturing system-induced variability rather than claiming universal hardware independence. For this purpose, it includes not only the parameter interactions on quality but also the influence of different printer architectures as a source of environmental variability. Furthermore, each filament type has different thermal conductivity, interlayer bonding, and mechanical deformation properties during the printing process. This makes it challenging to reliably predict quality outputs under a single model without accounting for material-specific nuances. Instead of attempting to physically overcome the inherent thermodynamic constraints of high-deformation polymers, this study aims to act as a diagnostic pre-press mechanism by reflecting and modeling this heterogeneity within the studied parameter space. This approach improves prediction reliability by supporting pre-process parameter decision-making rather than attempting to compensate for inherent physical fluctuations through the model alone.

In this context, the proposed Hybrid Multi-Material Quality–Ensemble System–Stacking–Gradient Boosting Regressor (HMQ-ES-Stack-GBR) model integrates heterogeneous and high-dimensional production data into an output-specific adaptive hybrid ensemble learning structure. To achieve this, the system is grounded in defined operational measurement standards and statistical data sanitization protocols, modeling each quality metric separately based on validated physical inputs. Ultimately, rather than serving as an inverse-optimization tool that automatically corrects physical flaws during the printing process, this proposed model offers a promising quality prediction framework for intelligent manufacturing systems within the scope of the studied dataset. It acts as a diagnostic tool, aiming to reduce time and raw material waste by identifying expected quality deviations prior to production. Additionally, to bridge predictive accuracy with optimal manufacturing performance, a multi-objective optimization framework utilizing three state-of-the-art meta-heuristic algorithms—Non-dominated Sorting Genetic Algorithm II (NSGA-II), Particle Swarm Optimization (PSO), and Grey Wolf Optimizer (GWO)—was integrated to systematically derive material-specific optimal parameter sets across the multi-output space.

Unlike previous studies that typically focus on a single material group, a single quality metric, or limited experimental datasets, the proposed framework combines heterogeneous material classes, output-specific data management, and hierarchical ensemble learning within a unified prediction architecture. The methodological novelty of this study lies in the integration of these components into a single framework specifically designed for multi-material FDM manufacturing environments. Unlike existing stacking or ensemble-based 3D printing studies in the literature that rely on a single global meta-learner for all outputs, the proposed framework introduces an output-specific data subclustering and error optimization pipeline. By training distinct meta-learners tailored to the unique physical behavior of each quality metric, the framework successfully prevents the multi-material variance of one property (e.g., highly elastic TPU modulus) from inducing noise into the prediction of another (e.g., rigid PLA roughness).

## 2. Materials and Methods

### 2.1. Experimental Design and Data Generation

The dataset is based on a systematic, large-scale experimental framework encompassing 1500 physical samples with 500 different parameter combinations and three replicates for each combination. This study utilized a material library of 10 polymers: PLA+, PETG, ABS, PLA-CF, TPU, PLA-phosphorus, PLA-transparent, PLA (standard), rPET, and Polypropylene (PP). Four different printing parameters and three levels for each parameter were selected: layer thickness (0.1, 0.2, 0.3 mm), infill density (20%, 50%, 80%), printing speed (30, 50, 70 mm/s), and infill pattern (zigzag, grid, triangles). Material type and printing parameters were defined as the main independent variables. To ensure the international validity of the mechanical tests, all samples were prepared and printed according to ISO 527-2 Type 4 standard dimensions [66]. All testing procedures were conducted in accordance with relevant international standards, and measurement devices were calibrated prior to testing to ensure consistency and repeatability. In this process, a strategic optimization was used to balance the distribution of 500 combinations. Operational constraints such as raw material costs and supply challenges were considered during this optimization. In this context, due to its widespread use in industry and multidimensional parameter interactions, PLA+ material formed the densest layer of the dataset with 240 different combinations (720 physical samples) encompassing 3 basic infill patterns (Zigzag, Grid, and Triangles) and 11 advanced infill geometries (Cubic, Quarter Cubic, Cross, Gyroid, Cubic Subdivision, Tri-Hexagon, Lightning, Cross 3D, Concentric, Octet, and Zigzag-Lines), resulting in a total of 14 infill configurations. For comparative analyses, PETG (92 combinations), ABS (27 combinations), PLA-CF (27 combinations), TPU (27 combinations), PLA-phosphorus (27 combinations), PLA-transparent (24 combinations), and standard PLA (18 combinations) were produced. For rPET and PP materials, which have cost and raw material constraints, the Taguchi experimental design method was used to reduce a full factorial matrix of 81 combinations into sets of 9 combinations each, representing the meaningful dataset. This deliberate optimization resulted in a heterogeneous material distribution, which reflects industrial prioritization but also introduces a class imbalance that was addressed during model training and validation. The experimental design was intentionally constructed to balance industrial realism and experimental reliability rather than to establish a fully balanced factorial structure across all materials and printer systems. Consequently, material-specific processing requirements were considered during specimen production to ensure printability, dimensional stability, and reproducible quality measurements.

To capture system-induced variability and the effect of ambient temperature on quality during production, Creality Ender-3 Pro (open case) and Creality K1 (closed case) printers were used simultaneously. This setup was designed to capture system-induced variability rather than to establish full hardware independence. PETG, PLA+, and PLA materials, which are sensitive to thermal gradients, were produced on both printer types (47 open-case, 453 closed-case combinations), and hardware variations were included in the data pool. The production of all other engineering filaments was carried out on the closed-case Creality K1 system to maintain thermal stability and minimize warping. This decision was made because engineering materials such as ABS, TPU, PP, and rPET are substantially more sensitive to thermal fluctuations, shrinkage, and warping effects than conventional PLA-based materials. Therefore, a closed-frame printing environment was selected to ensure reliable specimen production and experimental repeatability. To ensure printability and process consistency, material-specific nozzle and bed temperatures were maintained throughout the experimental campaign. PLA specimens were printed at 200 °C nozzle and 60 °C bed temperatures, PLA+ at 215 (open case)–220 °C (closed case) nozzle and 70 °C bed temperatures, PLA-phosphorus and PLA-CF at 220 °C nozzle and 70 °C bed temperatures, PETG at 240 (open case)–260 °C (closed case) nozzle and 75 (open case)–80 °C (closed case) bed temperatures, rPET at 280 °C nozzle and 80 °C bed temperatures, PLA-transparent at 200 °C nozzle and 60 °C bed temperatures, PP at 230 °C nozzle and 90 °C bed temperatures, TPU at 230 °C nozzle and 40 °C bed temperatures, and ABS at 250 °C nozzle and 100 °C bed temperatures. These temperatures were treated as controlled material-specific production conditions rather than independent experimental variables and were therefore not included as separate machine learning input features.

In the characterization phase, three physical replicas for each parameter set were tested separately. To establish clear operational measurement standards, a rigorous data sanitization protocol was applied prior to aggregation. Specifically, catastrophic print failures (e.g., incomplete geometry) and sensor-induced anomalies were defined as invalid zero values and removed. For the remaining numeric data, the Interquartile Range (IQR) method was utilized to identify and exclude statistically significant outliers (values falling below Q1–1.5×IQR or above Q3+1.5×IQR). Only these validated measurements were subsequently averaged to create the final 500-row ‘smart data matrix’, ensuring that physically meaningless data did not introduce noise into the model. This averaging strategy was employed to reduce experimental variance and improve measurement reliability, although it may also contribute to an optimistic estimation of predictive performance by reducing output variance within the training domain. Tensile tests were performed in an accredited laboratory using tensile testing equipment, and maximum stress, modulus of elasticity, and elongation at fracture data were recorded. Tensile tests were performed in an accredited laboratory using an MTS Criterion™ Series Model 45 Electromechanical Universal Testing Machine, and maximum stress, modulus of elasticity, and elongation at fracture data were recorded. To accommodate the distinct viscoelastic behaviors of the investigated material library under the ISO 527-2 Type 4 standard, crosshead separation speeds were tailored specifically per polymer group: elastomeric specimens (TPU) were tested at a crosshead speed of 50 mm/min to allow for stable elongation tracking, while all other rigid and functional polymer groups (PLA+, PLA, PETG, ABS, PLA-CF, PLA-phosphorus, PLA-transparent, rPET, and PP) were characterized at a standard speed of 5 mm/min.

Surface hardness values of the materials were determined using a PCE Instruments Shore D Digital Durometer, with data taken from five different points on the surface of each sample. All durometer characterization procedures, calibration checks, and multi-point sampling steps were strictly conducted in accordance with the ISO 868 and ASTM D2240 international standards for plastics indentation hardness measurement.

To quantify the effects of printing parameters on microscopic surface topography, the average roughness (Ra) parameter was characterized using a TR200 surface roughness tester. Ra values were recorded as the average of 3 measurements per sample. These multi-point and repeated measurement strategies were applied to enhance data reliability and minimize measurement noise.

**Figure 1:**
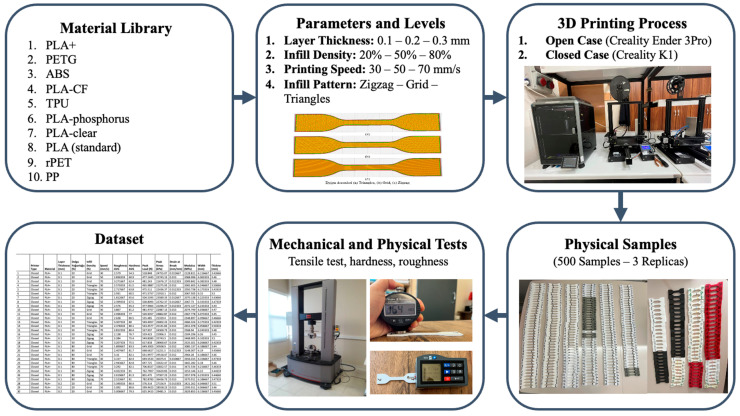
Overview of the experimental design, specimen production, test procedures, and dataset generation process.

The entire experimental workflow, from material selection and process parameter definition to specimen fabrication, mechanical and physical characterization, and final dataset generation, is presented in Figure 1. The figure also includes representative photographs of the actual ISO 527-2 Type 4 specimens produced in this study, together with the tensile testing system, Shore D hardness measurements, and surface roughness characterization procedures used during data acquisition.

Operational standards, equipment calibration procedures, and error control strategies implemented to ensure data consistency are detailed in Table 1. This structured framework defines the experimental metrics used throughout the study. While the HMQ-ES-Stack-GBR Hybrid ensemble model naturally captures physical and thermal variations between open- and closed-case printer architectures, it leverages these differences as a data source to learn complex, nonlinear interactions. By incorporating these system-induced variations and material-specific behaviors into the training phase, the model provides a framework for understanding quality deviations across the studied configurations. Consequently, the proposed approach demonstrates adaptability across different printer conditions, specifically within the constraints of the current experimental dataset, emphasizing its role in capturing system-induced variability rather than achieving full hardware independence.

### 2.2. Proposed Methodology: The HMQ-ES-Stack-GBR Framework

It should be noted that HMQ-ES-Stack-GBR is not introduced as a new machine learning algorithm. Instead, it represents a domain-specific hybrid ensemble framework that combines established machine learning models through a hierarchical stacking strategy. The originality of the framework stems from its adaptation to heterogeneous multi-material additive manufacturing data, output-specific preprocessing pipeline, and integrated quality prediction architecture.

Unlike conventional ensemble learning approaches that typically employ a single aggregation strategy, the proposed framework evaluates and integrates multiple ensemble mechanisms, including averaging, weighted averaging, and hierarchical stacking. Furthermore, OOF prediction-based meta-features are utilized to reduce information leakage and improve generalization performance. The framework was specifically designed to address the challenges associated with heterogeneous multi-material FDM datasets and simultaneous prediction of multiple mechanical and physical quality outputs.

In this study, a hybrid ensemble learning framework, the HMQ-ES-Stack-GBR model, is presented to predict the physical and mechanical quality characteristics of parts produced by FDM-based 3D printing processes. The developed framework addresses nonlinear relationships, material heterogeneity, and multi-output prediction challenges commonly encountered in FDM manufacturing datasets. By integrating comparative base learners, output-specific data management, and a hierarchical stacking mechanism, the model aims to provide stable and physically consistent predictions across different quality metrics. To ensure unbiased model development and evaluation, a two-stage validation strategy combining hold-out testing and cross-validation was adopted. The complete validation workflow is described below. The validation procedure consisted of two sequential stages. First, the complete dataset was divided into independent training (80%) and testing (20%) subsets using the hold-out method. The test subset was isolated from all model development procedures and was used exclusively for final performance evaluation. Second, only the training subset was subjected to 5-fold cross-validation. During this stage, OOF predictions were generated for each base learner (XGBoost, Random Forest, and GBR). These OOF predictions were subsequently used to construct the meta-feature matrix required for training the Level-1 meta-learner. After model development was completed, the final HMQ-ES-Stack-GBR framework was evaluated using the previously unseen hold-out test set. This two-stage validation strategy was adopted to reduce information leakage and obtain a more reliable estimate of predictive performance.

Ensemble learning approaches aim to improve prediction performance by combining multiple ML models. While single learners can produce results in certain data structures, they generally have limited generalization capabilities in complex and high-dimensional production data. Hybrid ensemble systems address these challenges by leveraging the complementary features of different learning algorithms. Linear models excel at capturing global trends, while tree-based and gradient boosting methods are better able to model nonlinear and interactive relationships. The HMQ-ES-Stack-GBR approach aims to produce more balanced and reliable predictions by systematically combining the predictive behaviors of these different model families within the studied dataset. Furthermore, in the proposed system, the dataset for each quality output is treated separately. Following the rigorous data sanitization protocol (e.g., IQR method) detailed in Section 2.1, modeling is performed using strictly validated observations specific to that output. This output-specific data management approach operationalizes our measurement standards, ensuring that physically meaningless anomalies do not skew the learning process. The proposed HMQ-ES-Stack-GBR framework is built on a two-level hierarchical stack learning architecture designed to capture system-induced variability. This structure consists of two main layers: Level-0 (basic learners) and Level-1 (meta-learners).

At Level 0, numerous regression models with different learning principles were evaluated. For comparative benchmarking, a broad set of baseline regression models was initially investigated, including Linear Regression, Ridge Regression, Lasso Regression, Elastic Net, K-Nearest Neighbors (KNNs), Decision Tree Regression (DTR), Support Vector Regression (SVR), Random Forest (RF), Gradient Boosting Regressor (GBR), and XGBoost. All baseline models were trained and evaluated under identical experimental conditions using the same training and testing partitions. This benchmarking stage was conducted to identify the most suitable learners for integration into the proposed hybrid ensemble framework.

As a result of the comparative analyses, XGBoost, RF, and GBR models, which effectively capture nonlinear relationships within this dataset, were selected as base learners in the stacking structure. To establish clear operational standards for model development, hyperparameter tuning for all base learners (XGBoost, RF, GBR) was systematically conducted using Grid Search optimization integrated with 5-fold cross-validation. During this training phase, OOF predictions were generated for each observation, ensuring that predictions were strictly derived from folds not seen during training, thereby minimizing the risk of information leakage. These OOF predictions obtained for each base learner were combined to form a meta-feature matrix for use in the meta-learning phase. OOF predictions refer to predictions generated for validation samples that were not used during the training of a particular cross-validation fold. In stacking architectures, these predictions are commonly employed to construct unbiased meta-features while minimizing information leakage between base learners and the meta-learner.

At Level 1, a meta-learning mechanism was implemented that takes the OOF predictions obtained from the base learners as input. In this phase, different integration strategies were evaluated: simple average, weighted average, and stack-based meta-modeling. Specifically, the GBR-based meta-learner was chosen as a model because it effectively learns nonlinear relationships between base models and patterns in prediction errors under the current experimental conditions. Since the meta-learner is trained only on cross-validation-based OOF predictions, the model’s performance consistency is preserved, and the risk of overfitting is managed within the training domain.

This hierarchical composition directly addresses a major gap in conventional additive manufacturing ensemble models, which typically rely on simple linear meta-regressors at Level-1. While linear aggregators fail to reconcile severe multi-material fluctuations, the proposed GBR meta-learner successfully maps the nonlinear error correlations among base models. Furthermore, by isolating each quality output into its own independent prediction pipeline, the framework prevents the distinct material variance of one target metric from distorting the prediction physics of another.

**Figure 2:**
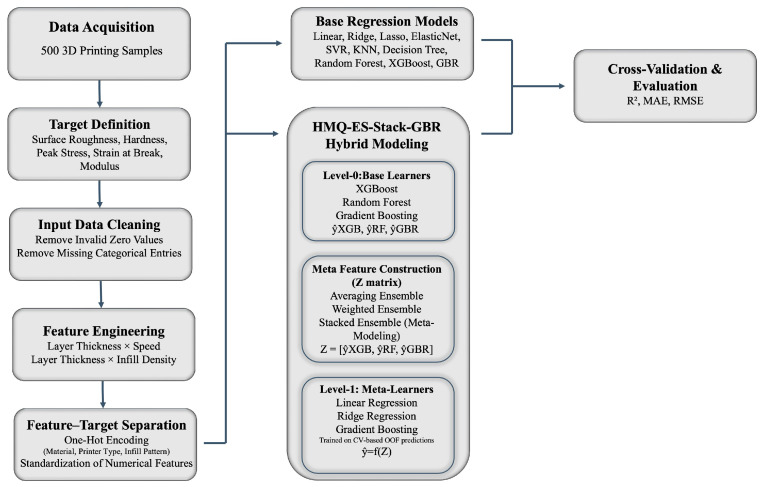
Developed HMQ-ES-STACK-GBR general workflow diagram and model architecture.

Thanks to this hierarchical structure, the HMQ-ES-Stack-GBR model combines the strengths of individual learners and demonstrates improved predictive performance within the studied dataset. All operational processes of this developed system, from data cleaning to the final prediction stage, are presented in detail in Figure 2. Ultimately, the meta-learner is engineered not to artificially ‘overcome’ the inherent physical variability of different polymers, but to accurately ‘reflect’ and predict these variations under the defined experimental conditions. By learning these complex error patterns operationally, the framework functions as a diagnostic tool that informs necessary pre-press parameter interventions for the investigated material groups.

All machine learning analyses were implemented in Python 3.13 using the Spyder integrated development environment (IDE). Data preprocessing, model development, cross-validation, ensemble learning, and performance evaluation procedures were performed using the Pandas (2.3.3), NumPy (2.2.6), Scikit-learn (1.7.2), and XGBoost (3.1.2) libraries.

Various hyperparameters were determined to improve the performance of the base learners used in the HMQ-ES-Stack-GBR model. The hyperparameter values used in this study were selected based on commonly used settings in the literature and preliminary trials. Performance evaluation of the models was carried out using the 5-Fold Cross Validation approach.

In the XGB model, the number of trees (n_estimators = 500), maximum depth (max_depth = 5), learning rate (learning_rate = 0.05), subsample rate (subsample = 0.9), and feature sampling rate (colsample_bytree = 0.9) were used. In the RF model, the number of trees was determined as 300 (n_estimators = 300). In the GBR model, a structure consisting of 300 trees (n_estimators = 300) was preferred. In the second level of the stacking architecture, GBR was used as a meta-learner, and the number of trees for the meta-learner was set to 200 (n_estimators = 200). For all models, the value random_state = 42 was used for randomness control during cross-validation. The hyperparameters used are presented in Table 2. OOF predictions obtained from the basic learners created using these parameters were combined to create a meta-feature matrix.

### 2.3. Multi-Objective Process Optimization Framework

The hybrid ML model developed within the scope of this study was not only used to predict quality outcomes but also was evaluated as a decision support mechanism for determining optimum production parameter combinations. FDM-based 3D printing processes have a complex and nonlinear structure that occurs under the simultaneous interaction of many production parameters such as layer thickness, printing speed, infill density, and infill pattern. For this reason, classical deterministic optimization methods cannot show sufficient performance in high-dimensional and nonlinear solution spaces. On the other hand, heuristic optimization algorithms can reach global optimum regions more effectively in complex engineering problems.

Within the scope of this study, NSGA-II, PSO, and GWO algorithms were used to compare different optimization behaviors. In the optimization processes, the hybrid machine learning model was used as an objective function generator, and quality outcomes were estimated for different production parameter combinations. Thus, it was aimed to determine the optimum production parameters without performing physical production.

Optimization analyses were carried out separately for closed and open system printers. Thus, it is aimed to reduce the effect of thermal and environmental differences that may arise from the printer architecture on the optimization results. While TPU, PLA+, PLA, PLA-CF, ABS, PETG, rPET, and PP filaments are evaluated in closed system optimization analyses, in open system analyses, only PLA, PLA+, and PETG filaments that can be produced safely in open system printers were taken into account.

#### 2.3.1. NSGA-II Algorithm

NSGA-II is one of the evolutionary optimization algorithms widely used in multi-objective optimization problems [69]. By using a population-based structure, the algorithm can simultaneously search for more than one optimum solution region in the solution space. It can produce effective results, especially in engineering problems where conflicting target functions exist. In the NSGA-II algorithm, solution candidates are evaluated with the non-dominant ranking approach, and Pareto-optimum solution regions are determined (Equation (1)). The general multi-objective optimization structure of the algorithm is expressed as follows:

(1) $$min\;F(x)=\left[f_{1}\left(x\right),f_{2}\left(x\right),\ldots ,f_{n}(x)\right]$$

Here, ***F(x)*** is the multi-objective optimization function; $f_{n}$(x) represents each target function. Within the scope of this study, different printing parameter combinations were evaluated using the NSGA-II algorithm, and optimum solution regions were determined in terms of quality outputs. The algorithm has been used especially to investigate large parameter spaces.

#### 2.3.2. PSO Algorithm

PSO is one of the swarm-intelligence-based heuristic optimization algorithms [70]. The algorithm was developed inspired by the collective movement behavior of flocks of birds. In the PSO algorithm, each solution candidate is defined as a particle, and the particles move in the solution space and try to reach optimum regions (Equations (2) and (3)).

In the PSO algorithm, the velocity update process of particles is expressed by the following equation:

(2) $$v_{i}^{t+1}=wv_{i}^{t}+c_{1}r_{1}(p_{i}-x_{i}^{t})+c_{2}r_{2}(g-x_{i}^{t})$$

The new position of the particle is calculated as follows:

(3) $$x_{i}^{t+1}=x_{i}^{t}+v_{i}^{t+1}$$

Here $v_{i}$ represents the particle speed, $x_{i}$ represents the particle position, $p_{i}$ represents the individual best solution and g represents the global best solution. Within the scope of this study, optimum combinations of layer thickness, printing speed, and filling density parameters were investigated using the PSO algorithm. It has been observed that the algorithm creates more stable optimum regions in many filament types.

#### 2.3.3. GWO Algorithm

GWO is a heuristic optimization algorithm inspired by the social hierarchy and hunting behavior of gray wolves [71]. In the algorithm, solution candidates are modeled as a wolf pack, and the best solutions are represented by alpha, beta, and delta wolves (Equations (4) and (5)).

In the GWO algorithm, the updating of solution candidates is expressed by the following equations:

(4) $$D=|C\cdot X_{p}(t)-X(t)|$$

(5) $$X(t+1)=X_{p}(t)-A\cdot D$$

Here, X plider represents the wolf position; A and C represent the coefficient vectors that govern the containment of the prey. Within the scope of this study, optimum production parameter combinations for different filament types were investigated using the GWO algorithm. It has been observed that the algorithm produces successful results, especially in filaments that exhibit elastic behavior.

#### 2.3.4. Objective Function and Optimization Criteria

In the optimization process, the objective function (Equation (6)) was created using the quality outputs predicted by the hybrid machine learning model. The objective function aims to minimize surface roughness and maximize hardness, maximum stress, elongation at break, and modulus of elasticity.

The optimization score used in this context is defined as follows:

(6) $$Score=-R_{a}+H+\frac{\sigma_{max}}{1000}+100\varepsilon_{b}+\frac{E}{100}$$

Here:

Ra: surface roughness;
H: hardness;
$\sigma_{max}$: maximum stress;
$\varepsilon_{b}$: elongation at break;
E: modulus of elasticity.

The scaling factors used in Equation (6) (i.e., division by 1000 for maximum stress, multiplication by 100 for elongation at break, and division by 100 for modulus of elasticity) were introduced solely to balance the numerical magnitudes of the quality metrics within the aggregated objective function. Since the investigated outputs are expressed in different physical units and exhibit substantially different value ranges, these fixed scaling factors prevent variables with larger numerical magnitudes from dominating the optimization score. They are not empirically optimized weighting coefficients but deterministic scaling constants intended to preserve the engineering interpretation of each quality metric while ensuring numerical stability during optimization. 

In the optimization analyses, layer thickness, printing speed, and infill density were considered as continuous variables, while the infill pattern was evaluated as a categorical parameter. In addition, each filament type was optimized separately within its own parameter range. Thus, the differences in the mechanical and physical behavior of different materials were prevented from suppressing the optimization results. This approach aims not only to predict quality outputs but also to determine optimum printing parameters before physical production takes place. Thus, the developed hybrid system has been transformed into an integrated decision support structure capable of performing quality prediction and process optimization simultaneously.

## 3. Results

### 3.1. Dataset Analysis

**Figure 3:**
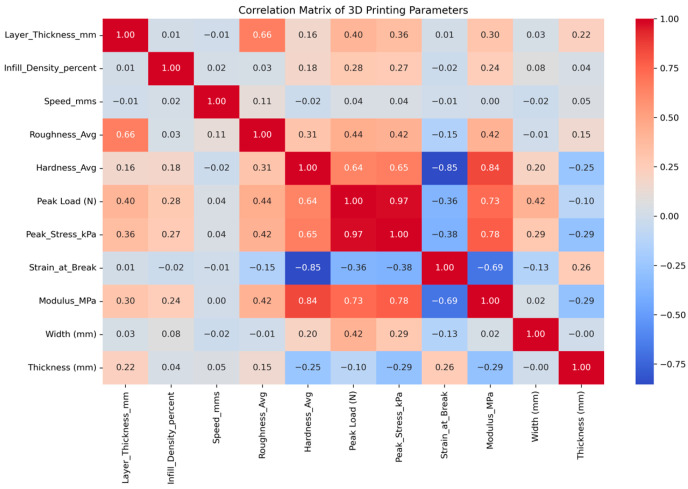
Pearson correlation matrix showing the relationships between mechanical and physical properties and 3D printing parameters used in quality assessment.

When examining the results of the Pearson correlation analysis, which quantitatively reveals the relationship between the data obtained from the experimental process (Figure 3), a clear hierarchical interaction is observed between 3D printing parameters and mechanical outputs, in particular, the strong positive correlation (r = 0.66) between layer thickness, which directly affects the production geometry, and surface roughness supports the step effect theory in the literature. The high correlation between the rigidity and surface hardness of the samples (r = 0.84) indicates that, in terms of mechanical performance, the structural resistance of the material directly affects its surface properties. However, there appears to be a suppressive effect of increased hardness on ductility. The strong negative correlation between hardness and elongation at fracture (r = −0.85) confirms that efforts to maximize mechanical strength increase the brittleness of the material within this dataset. On the other hand, the ineffective profile of the printing speed on all outputs reveals that speed is not a significant determinant within the selected parameter range. At the same time, the moderate effect of mechanical strength on the infill ratio (r = 0.25) shows that the final performance is shaped not only by the infill amount but also by the synergy of variables such as infill pattern and material type. This shows that there are only weak linear relationships between some process parameters, such as speed and infill ratio, and quality outputs under the current experimental conditions. In contrast, the prediction performance of the proposed HMQ-ES-Stack-GBR model reveals that these parameters have indirect but measurable effects on quality outputs through nonlinear and multivariate interactions. This suggests that the hybrid ensemble learning approach is better suited than univariate linear statistical methods for capturing the complex, multidimensional nature of the FDM process within the studied dataset.

**Figure 4:**
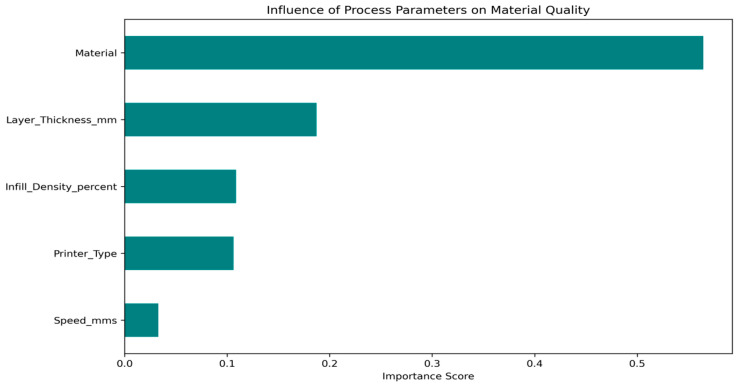
Importance levels of process parameters on output quality.

The feature significance levels obtained through the HMQ-ES-Stack-GBR model (Figure 3) present the hierarchical structure that determines the quality in the 3D printing process within the scope of the studied dataset. According to the analysis results, material type stands out as the most dominant factor, accounting for more than half of the total variation with approximately 0.55 points, suggesting that characteristic differences in polymer structure exert a more substantial influence on outcomes than the selected operational parameter changes. The second most significant parameter is layer thickness, while the effects of infill ratio and printer type on quality remained similar. Another noteworthy point is that printing speed has a relatively low significance score compared with other variables. This parallels the findings in the previous correlation matrix (Figure 3). It confirms that the chosen speed range has a secondary effect on mechanical performance under the current experimental conditions. This dominance of material characteristics indicates that data-driven predictive models primarily reflect structural and thermodynamic variations rather than physically overcoming them. Consequently, this statistical observation reinforces the intended role of the proposed architecture as an accurate diagnostic tool rather than an automated correction mechanism. To quantify the relative contribution of each independent variable to the global printing quality, a centralized feature importance analysis (Figure 4) was conducted. The feature importance scores were derived directly from the Level-1 GBR meta-learner of the proposed HMQ-ES-Stack-GBR framework. Specifically, the model utilizes the Mean Decrease in Impurity (MDI)—also known as Gini importance—recalculated across all ensemble nodes within the trained gradient-boosted decision trees. By measuring the average reduction in variance brought by each feature across all structural splits, this method provides a robust, multi-output integrated insight into how heavily the framework relies on material types versus operational machine parameters during the meta-learning prediction phase.

To evaluate experimental repeatability, the statistical characteristics of the replicate measurements were analyzed using the coefficient of variation (CV). The mean CV values were 2.89% for peak stress, 3.00% for strain at break, 2.83% for modulus, 2.95% for hardness, and 11.22% for surface roughness (Table 3). The relatively low variation observed in tensile and hardness measurements indicates good measurement consistency across replicates, whereas the higher variability observed for surface roughness reflects the inherent stochastic nature of layer-by-layer deposition and local surface topography variations in FDM processes. Following the predefined validation and outlier-removal procedures, validated replicate measurements were averaged to construct the final dataset used for model development.

### 3.2. Baseline Models and Comparative Evaluation

The ability of the developed HMQ-ES-Stack-GBR hybrid ensemble architecture to predict 3D printing quality parameters was evaluated on a dataset of 500 physical samples obtained from 10 different material types (TPU, PLA-CF, rPET, etc.) using multidimensional statistical metrics and a comparative analysis. The model’s performance was tested using a 5-fold cross-validation method with coefficient of determination (R^2^), mean absolute error (MAE), and root mean square error (RMSE) metrics. Empirical findings reveal that the proposed hierarchical stacking structure demonstrates improved predictive performance compared with the evaluated model in this study under the same experimental conditions across all quality outputs.

Examining the numerical results, we see that the HMQ-ES-Stack-GBR model demonstrates prediction accuracy for both mechanical and physical properties within the studied dataset, achieving R^2^ values of 0.999 for elongation at break, 0.999 for maximum stress, and 0.994 for modulus of elasticity. Similarly, the prediction of hardness showed accuracy (R^2^ = 0.991). Surface roughness, one of the most complex parameters to model among the physical properties, also exhibited a consistent performance (R^2^ = 0.936).

The R^2^ ratios of 0.93 and above exhibited by the model reveal that the variability within the experimental outputs is interpreted with high accuracy by the model. This suggests that the model is able to capture variability arising from material heterogeneity effectively within the training domain. Table 4 below details the performance metrics for each target variable. The absorption of variance in the experimental data by the model is reflected in R^2^ values greater than 0.93, providing an upper-bound performance estimate for the examined parameter space.

**Figure 5:**
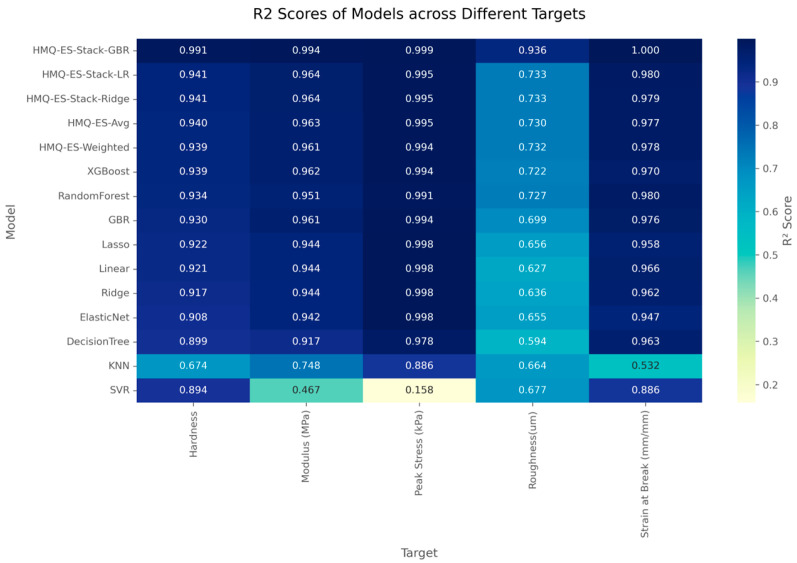
Comparison of R^2^ metrics of traditional ML, ensemble learning models, and the proposed HMQ-ES-Stack-GBR model.

Comparative analyses reveal that the proposed HMQ-ES-Stack-GBR architecture exhibits improved predictive performance across all quality outputs, compared with the baseline models evaluated in this study (Figure 5). Examining the performance differences observed in Figure 5 demonstrates that traditional singular ML algorithms such as KNN and SVR lag behind the HMQ-ES-Stack-GBR architecture within the current dataset. This is thought to be due to the KNN algorithm losing its discriminative power because of its distance metric-based decision-making in high-dimensional feature spaces, and SVR having limited flexibility in modeling nonlinear, multivariate interactions. In contrast, the proposed hierarchical ensemble architecture, thanks to its stacking structure that progressively optimizes the error models of weak learners, is able to represent complex nonlinear relationships in a more stable manner under the defined experimental conditions. Particularly in surface roughness estimation, while the HMQ-ES-Stack-LR model, the closest success metric, maintained an R^2^ value of 0.733, the proposed architecture achieved a value of 0.936, demonstrating the effectiveness of the hybrid framework in capturing complex surface variations within this dataset. A similar situation was observed in the estimation of modulus of elasticity (R^2^ increase: from 0.964 to 0.994) and elongation at break (R^2^ increase: from 0.980 to 0.999). This performance improvement is based on a hierarchical processing process. In this context, predictions from the XGBoost, RF, and GBR algorithms located in the Level-0 layer are refined by a GBR-based meta-learning algorithm in the Level-1 layer and transformed into the final result. The meta-learner captures nonlinear error relationships among base learners, enabling improved representation of complex data patterns and enhancing predictive performance under controlled laboratory conditions.

Another factor influencing the model’s stability is the “output-specific data management” approach applied to each quality output. As defined by the operational measurement standards in Section 2.1, statistically identified outliers (via the IQR method) and catastrophic physical failures specific to each output parameter have been systematically eliminated. Additionally, by using OOF predictions during training, information leakage is prevented, thus minimizing the risk of overfitting within the experimental dataset.

In conclusion, all these numerical findings and error distribution analyses confirm that the HMQ-ES-Stack-GBR model offers consistency in representing the industrial complexity of heterogeneous manufacturing data within the studied parameter space, from elastomeric materials to high-strength composites. The findings suggest that the proposed framework provides a promising diagnostic tool for pre-print quality estimation, supporting decision-making processes by identifying expected quality deviations prior to production.

### 3.3. Overall Performance Evaluation Across Mechanical and Physical Outputs

**Figure 6:**
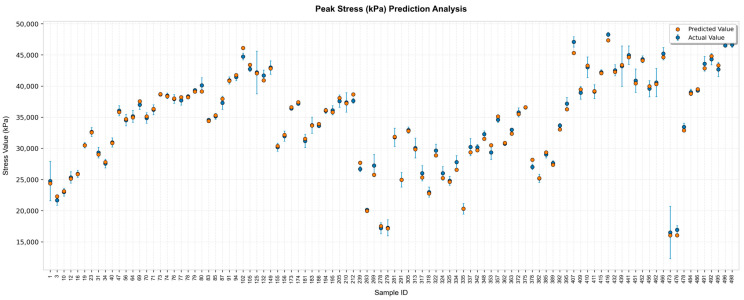
Comparative analysis of experimental vs. predicted values for tensile testing (peak stress).

**Figure 7:**
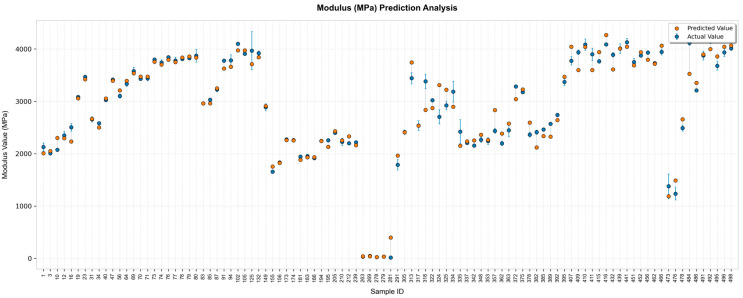
Comparative analysis of experimental vs. predicted values for tensile testing (modulus).

**Figure 8:**
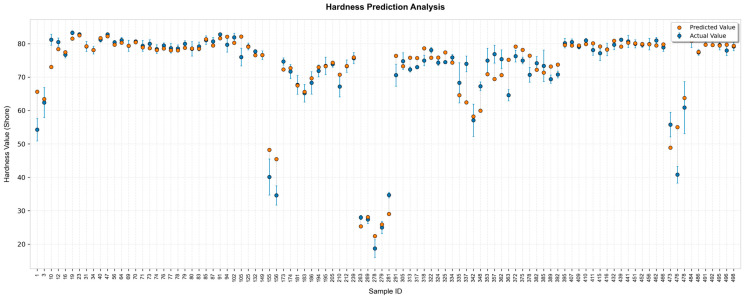
Comparative analysis of experimental vs. predicted values for hardness.

**Figure 9:**
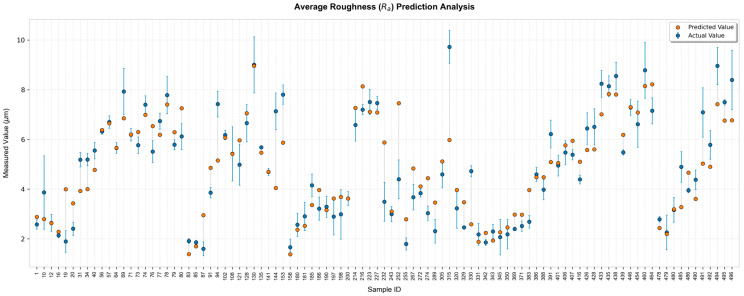
Comparative analysis of experimental vs. predicted values for roughness.

The predictive performance of the HMQ-ES-Stack-GBR model on test data is presented in Figure 6, Figure 7, Figure 8 and Figure 9, where the model’s predictions are compared with actual measurement values. Specifically, peak stress is shown in Figure 6, modulus results in Figure 7, hardness values in Figure 8, and roughness results in Figure 9. These graphs facilitate a comparative analysis between experimental results obtained from various randomly selected material types and sample identities from the dataset and the model’s predictions. In these visualizations, blue dots represent actual experimental measurements performed in a laboratory setting, while orange dots represent the model’s predicted values for the respective materials.

The peak stress results shown in Figure 6 demonstrate that the model exhibits a trend paralleling the experimental data across a wide stress range. For different material groups spanning values from 15,000 to above 45,000 kPa, the predicted values show varying degrees of deviation from the measured data within the studied dataset.

The modulus results presented in Figure 7 reflect the model’s capability to estimate the stiffness characteristics of the materials. For samples with values ranging between 1000 and 4000 MPa, an agreement between predictions and experimental data is observed under controlled laboratory conditions. For extreme cases such as TPU with low modulus values, the model captures the overall trend, although deviations increase to some extent.

The hardness results given in Figure 8 reveal the model’s performance in predicting surface resistance characteristics. Across samples exhibiting a wide distribution on the Shore D scale, the model produces results that are aligned with the trends of experimental measurements. In cases where sudden changes in hardness values occur, the model is able to follow the trend, although deviations may be present.

The average surface roughness results presented in Figure 9 indicate the model’s ability to estimate surface quality parameters. For roughness values ranging from 1 to 9 µm, the model shows a general agreement with the experimental data. Compared with other mechanical properties, this parameter exhibits higher variability; however, the model reflects the main trends, with deviations observed.

### 3.4. Model Validation and Predictive Performance Analysis

**Figure 10:**
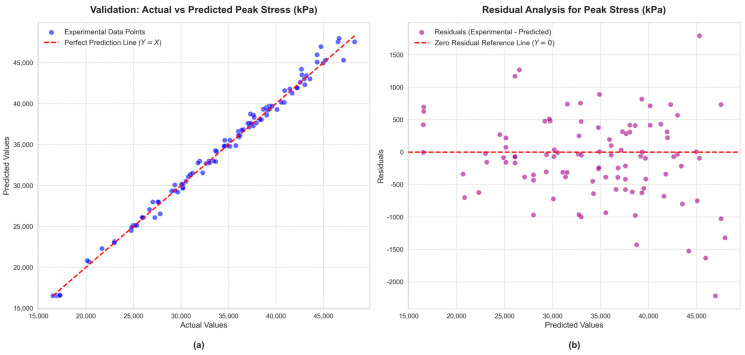
Performance validation of the HMQ-ES-Stack-GBR hybrid model for peak stress: (**a**) correlation between actual and predicted values, (**b**) distribution of residuals relative to predicted values.

**Figure 11:**
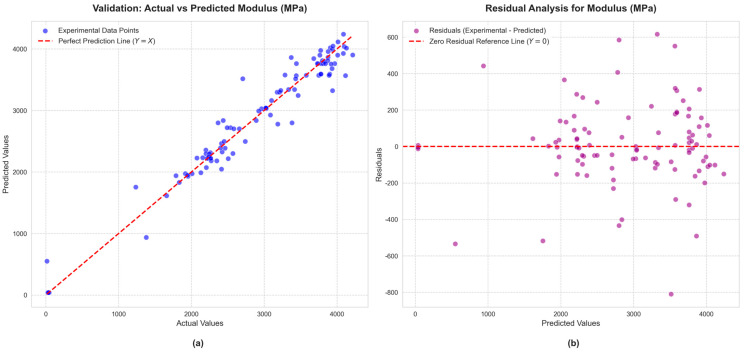
Performance validation of the HMQ-ES-Stack-GBR hybrid model for modulus: (**a**) correlation between actual and predicted values, (**b**) distribution of residuals relative to predicted values.

**Figure 12:**
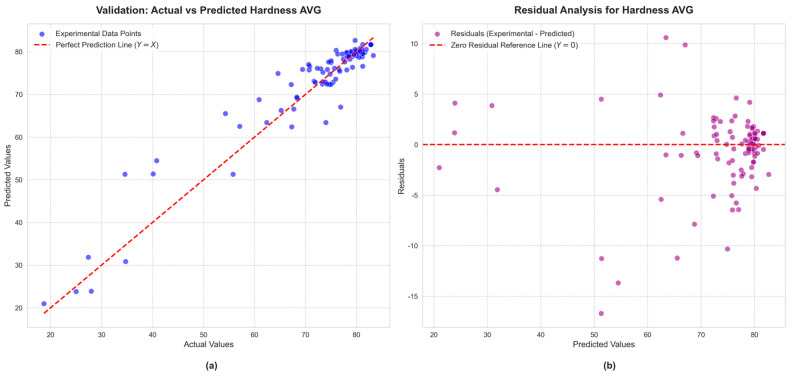
Performance validation of the HMQ-ES-Stack-GBR hybrid model for hardness AVG: (**a**) correlation between actual and predicted values, (**b**) distribution of residuals relative to predicted values.

**Figure 13:**
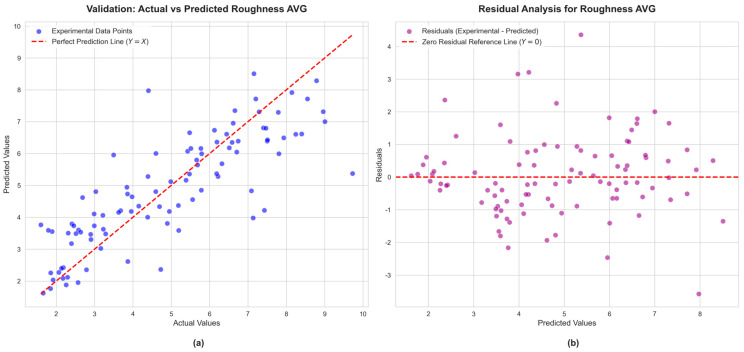
Performance validation of the HMQ-ES-Stack-GBR hybrid model for roughness AVG: (**a**) Correlation between actual and predicted values, (**b**) Distribution of residuals relative to predicted values.

The predictive performance of the HMQ-ES-Stack-GBR hybrid model across the output parameters was evaluated using an internal 80/20 data split, and the results are presented in Figure 10, Figure 11, Figure 12 and Figure 13. Correlation plots (a) and residual distributions (b) provide a general overview of the model’s diagnostic capability within the current experimental setup.

For Peak Stress (Figure 10), the model shows agreement with experimental data within the range of 15,000–48,000 kPa, with residuals largely distributed around the zero axis. A similar trend is observed for the Elastic Modulus (Figure 11), where the predicted values follow the variation within the 0–4200 MPa range without a systematic bias under the defined laboratory conditions.

In the analysis of Mean Hardness (Figure 12), the data points tend to cluster around the ideal fit line, particularly in regions of higher hardness. For mean roughness (Figure 13), a relatively higher dispersion is observed, which can be attributed to the inherent variability of surface characteristics in AM; nevertheless, the model reflects the overall trend with an alignment to experimental measurements.

Across all parameters, the random distribution of residuals suggests that the hybrid architecture is capable of representing complex nonlinear relationships within the training domain. Considering the internal validation approach and potential imbalances in the dataset, the model can be regarded as providing consistent performance within the examined parameter space. Furthermore, future research focusing on external validation across independent datasets is required to assess the generalizability of these findings beyond the current experimental context.

### 3.5. Statistical Significance Analysis

**Figure 14:**
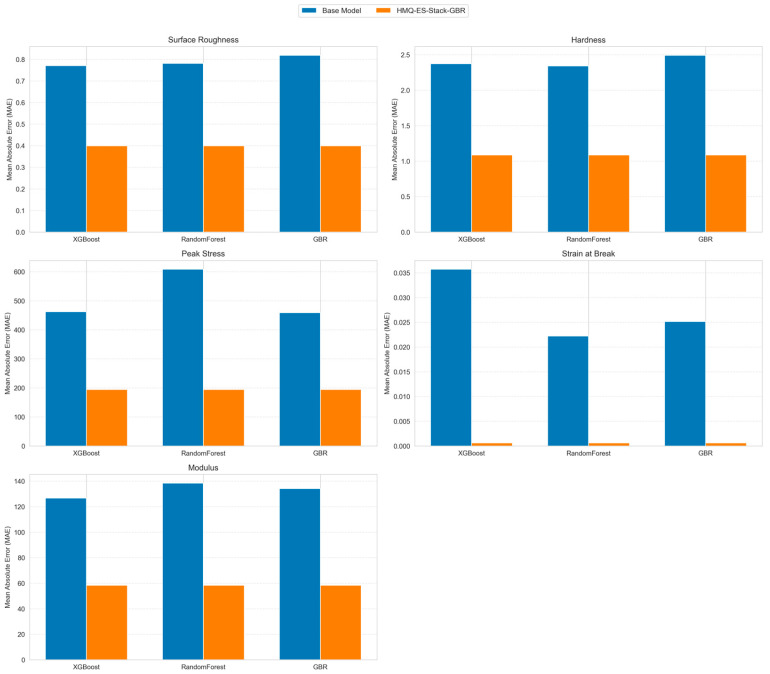
Comparison of mean absolute errors between baseline models (XGBoost, Random Forest, and GBR) and the proposed HMQ-ES-Stack-GBR across all target variables.

The predictive performance of the proposed HMQ-ES-Stack-GBR model was evaluated in comparison with XGBoost, Random Forest, and GBR methods under the same experimental conditions. To ensure the comparison was unbiased, all models underwent the same data splitting, feature engineering, and hyperparameter optimization preprocessing pipeline. Examining the error analysis results presented in Figure 14, it is observed that the proposed model achieved lower MAE values than the base models for surface roughness, hardness, peak stress, strain at break, and modulus within the current dataset. The decrease in error rate, particularly in strain-at-break predictions, demonstrates the hybrid architecture’s effectiveness in modeling complex datasets within the training and testing domains. To rigorously validate these performance improvements, paired *t*-tests and Wilcoxon signed-rank tests were performed, confirming that the reduction in error is statistically significant (*p* < 0.05) for all target variables.

To test the statistical validity of this improvement in numerical error values, paired *t*-tests and Wilcoxon signed-rank tests were applied. According to the analysis results in Table 5, the *p*-values calculated for all target variables remained below the 0.05 significance threshold. These results indicate that the performance improvement offered by the HMQ-ES-Stack-GBR model is a statistically significant difference compared with the base models under the evaluated conditions. These findings demonstrate that the proposed method produces more consistent predictions compared with the evaluated basic algorithms within the examined parameter space.

### 3.6. Material-Specific Performance Analysis

**Figure 15:**
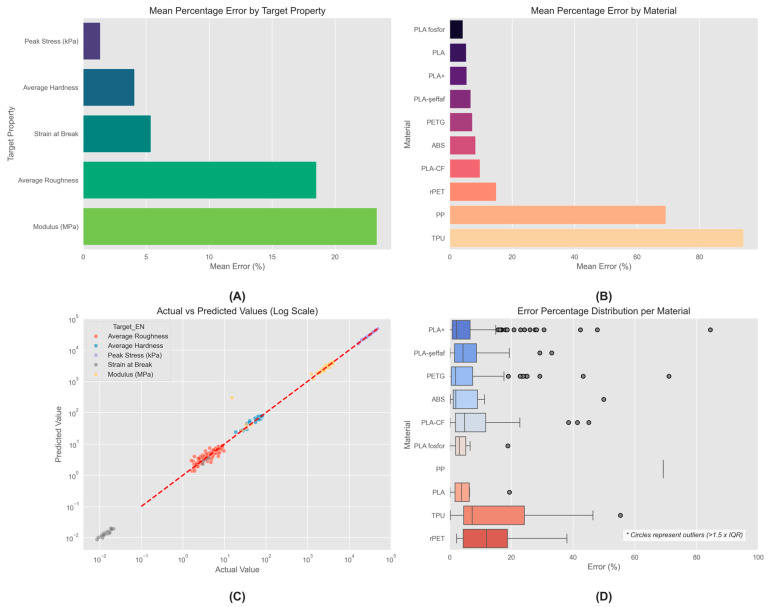
Multidimensional error and performance analysis of the HMQ-ES-Stack-GBR model: (**A**) mean percentage error (MPE) distribution according to target quality parameters; (**B**) prediction accuracy of the model according to 10 different filament types used; (**C**) logarithmic scale agreement between actual and predicted values of different output sizes (micro and macro); (**D**) box plot analysis showing material-based error variations and outliers.

The predictive performance of the developed HMQ-ES-Stack-GBR model is evaluated across various test cases; its predictive consistency and error profile assessments in test data for both material groups and quality outputs are detailed in Figure 15. These results show that the model’s strength lies in its ability to reflect physical anomalies and variability that develop throughout the manufacturing process, within the limitations of the current experimental design.

In Figure 15A, the Peak Stress estimates, in particular, are identified as the area where the model is most stable, according to both the sensitivity analysis and the mean percentage error (MPE) distribution results, reflecting the model’s adaptability to the relevant parameter under laboratory conditions. The effect of structural parameters such as layer thickness and infill ratio on mechanical strength is captured by the model, showing that the deviations in the regression results remain within a narrow range within the studied parameter space. In contrast, the increase in error in the Modulus parameter is thought to be due to the sensitivity of the modulus of elasticity to instantaneous physical changes such as interlayer cooling rates and thermal gradients.

Figure 15B, showing material-based performance data, indicates the decisive role of polymer type in prediction accuracy. The relatively low error rates in rigid thermoplastics such as PLA, PLA+, and PETG stem from their stable thermal behavior. In filaments such as TPU and PP, which exhibit high elasticity and a tendency towards thermal shrinkage, higher error rates have been observed. This is a consequence of the structural complexity of the prediction process, driven by the nonlinear deformation properties of these materials. Crucially, rather than artificially overcoming or masking these inherent thermodynamic constraints, the model reflects this physical behavior. This supports the model’s utility as a diagnostic indicator, warning operators that these specific high-deformation materials require targeted pre-press parameter optimization.

Furthermore, it is critical to acknowledge that this variance in predictive performance across different materials and targets is governed by a dual combination of material-specific physical behaviors and inherent dataset class imbalance. From a physical perspective, highly elastic or high-deformation polymers like TPU and PP exhibit severe nonlinear viscoelastic behaviors, thermal shrinkage, and sensitivity to instantaneous physical fluctuations (such as interlayer cooling rates) that are fundamentally more challenging to model than the stable thermal profiles of rigid thermoplastics like PLA or PETG. Concurrently, this lower predictive accuracy is structurally compounded by the class imbalance within the experimentally generated dataset, where dominant materials like PLA+ represent the densest layer (240 combinations), while materials like PP and rPET are significantly underrepresented (9 combinations each) due to Taguchi optimization and raw material constraints. Because the framework learns directly from the frequency of available data, the overrepresentation of conventional filaments inherently grants the model stronger feature space mappings for rigid polymers, whereas the sparse data density for elastic filaments limits the meta-learner’s ability to fully mitigate cross-property noise, thereby leading to the observed disparities in Figure 15B.

Figure 15C shows the logarithmic distribution of actual values and predictions, illustrating the model’s performance across different scales. Indeed, the proposed architecture exhibits a wide operating range, processing micro $10^{-2}$ values, such as elongation at break, with equivalent accuracy, and macro $10^{5}$ quantities, such as maximum stress. The data are observed to align around the ideal (y = x) line for the majority of the samples. This demonstrates that the model produces consistent results across a wide range of operating parameters within the examined domain.

Figure 15D shows a box plot of the system’s stability, revealing the extent to which the error distribution lies within a controlled band. The fact that the median errors do not exceed the 10% threshold in most material groups suggests the robustness of the proposed architecture within the current setup. Even in the PLA-clear and PLA-phosphorus examples, which differ in their functional characteristics, the rarity of outliers is consistent with the hierarchical structure’s ability to manage variance from heterogeneous datasets.

### 3.7. Printer System-Based Performance Analysis

**Figure 16:**
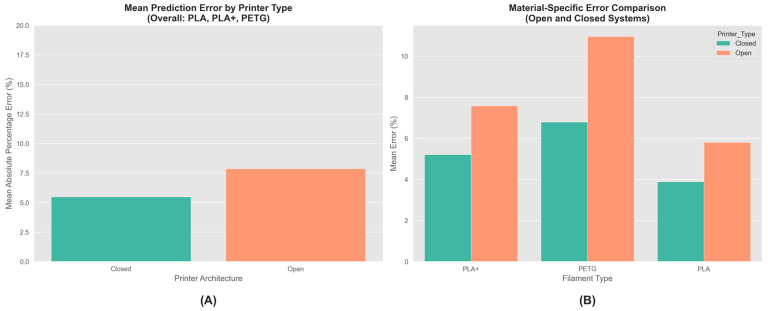
Performance analysis of open and closed 3D printers on PLA, PLA+, and PETG materials: (**A**) General prediction error distribution, (**B**) Comparison of material-based prediction error distribution.

In this study, a comparative analysis was conducted on PLA, PLA+, and PETG filaments, which are commonly produced in both open-case and closed-case systems, to determine the effect of hardware variations on model stability (Figure 16). This approach follows a method that stabilizes material variables to measure the impact of printer architecture and thermal environmental control on quality within the investigated setup. The presented average error results were compiled by filtering the test data of the HMQ-ES-Stack-GBR model.

Figure 16A shows that the measured average error margin is 5.48% in closed systems and 7.86% in open systems. These results suggest that the preference for open systems negatively impacts the error margin by approximately 43% for these specific materials. Open systems are sensitive to sudden temperature changes and uncontrolled airflow depending on ambient conditions. Stochastic noise (physical uncertainty) generated by these processes can complicate the model’s learning process, thus limiting the achievable accuracy compared with controlled thermal environments.

Figure 16B shows that the effect of thermodynamic properties on model performance is evaluated with material-specific analyses. These results indicate that the model partially accounts for the physical variations induced by the printing environment. PETG, known for its sensitivity to thermal shrinkage and interlayer cooling rate, exhibited higher average errors in open systems compared with closed systems. The inclusion of these micro-deviations in the prediction process demonstrates that the system can reflect complex physical processes to a certain extent. Furthermore, the results show that printer-related variables do not cause a significant deviation in prediction accuracy for PLA and PLA+ filaments. This resistance suggests that the HMQ-ES-Stack-GBR architecture manages mechanical noise and provides a consistent prediction baseline under the current experimental design. Furthermore, by material performance, the framework highlights hardware-induced quality drops rather than artificially overcoming them.

### 3.8. Multi-Material Process Optimization and Design Guidelines

In the optimization process, layer thickness, printing speed, infill density, and infill pattern parameters were evaluated. Within the objective function, the goal was to minimize surface roughness and maximize hardness, maximum tensile strength, elongation at break, and modulus of elasticity. Accordingly, each filament type was optimized separately within its own parameter range, and material-specific optimum parameter combinations were determined. Analysis of the results reveals that optimum production parameters showed significant differences depending on the type of material used. This indicates that material properties have a decisive effect on mechanical and physical quality outputs in FDM processes. Furthermore, it was observed that there is no single optimum parameter region common to all materials, and each filament type exhibits different production behavior. When examining the results obtained with TPU filament, it was determined that the zigzag infill pattern, combined with medium layer thickness and high infill density, produced the highest optimization scores. Due to the high deformation capacity and elongation at break values of TPU material, higher scores were obtained in the optimization outputs compared with other filament types. However, it was observed that the surface roughness values in TPU filament were higher than in some other filament types. It has been determined that combinations of low layer thickness and high filler density improve the mechanical performance of PLA and PLA+ filaments. High stiffness and modulus of elasticity values were obtained, particularly in PLA+ filament. In PLA-CF filament, lower surface roughness values were observed due to the effect of carbon fiber reinforcement, resulting in advantageous surface quality. In ABS filament, high filler density and medium-high printing speeds were found to have positive results in terms of mechanical strength. In PETG filament, it was determined that the zigzag filler pattern produced more balanced results between mechanical performance and surface quality. Lower mechanical performance values were obtained in rPET and PP filaments compared with other engineering filaments, and this was assessed to be due to the structural properties of the materials. When comparing the performance of the optimization algorithms, it was observed that the PSO algorithm produced higher optimization scores in most material types. The GWO algorithm yielded more successful results, especially in TPU, PLA+, and PLA-CF filaments. Although the NSGA-II algorithm produced competitive results, it lagged behind other algorithms in some materials. These results indicate that the PSO algorithm can identify more stable optimum regions in the current parameter space. Table 6 presents the optimum parameter combinations based on the material and their corresponding quality outputs. Overall, the results show that the material-based optimization approach offers more realistic and applicable results in FDM processes. Furthermore, the fact that the optimum parameter combinations vary depending on the material indicates that using material-specific optimization strategies instead of a universal parameter set is more appropriate in production processes.

## 4. Discussion

Unlike traditionally known ML models in the literature on 3D printing processes, this study comprehensively addresses the prediction of mechanical and physical test results obtained depending on 3D printing parameters using a hybrid HMQ-ES-Stack-GBR model. A dataset consisting of 500 physical samples covering 10 different material groups has enabled the model to exhibit adaptability across a wide range of materials, from highly elastic elastomers like TPU to carbon fiber-reinforced PLA-CF composites. Numerical results reveal improved predictive performance of the proposed stacking-based architecture compared with baseline models under the same experimental conditions, particularly in a parameter with high physical complexity, such as surface roughness. While the closest fixed-weight models achieved 73.3% performance, the developed model achieved 93.6%. This significant improvement is primarily due to the GBR-based meta-learner positioned at the Level-1 layer, which optimizes error correlations and material-based features across the basic learners (XGBoost, RF, GBR). In addition, the inclusion of both open- and closed-case printer systems in the process enabled the model to capture system-induced variability within the dataset and allowed partial representation of thermal and environmental effects on output quality. The “output-specific data management” approach allowed the model to represent industrial complexity with accuracy. By applying the strict operational measurement standards and statistical sanitization (e.g., the IQR method) defined in Section 2.1, the model systematically eliminated physically meaningless anomalies. All these findings demonstrate that the proposed hybrid architecture shows promising capability as a diagnostic quality prediction framework within the studied dataset, which minimizes raw-material waste and increases pre-press accuracy in smart manufacturing facilities. Crucially, while the system does not physically overcome the inherent thermodynamic limitations of certain polymers, it functions as a diagnostic tool by accurately reflecting these variations to inform required pre-press parameter adjustments.

A comparative analysis of the current study with similar research in the literature (Table 7) presents the contributions of the proposed model and methodology. A current literature review reveals that many studies aiming to predict 3D printer outputs rely on small datasets. Since models trained on small datasets will also have a small test set, there may be limitations when evaluating success metrics—for example, Selvan et al. The small sample size of 27 samples in the current study (2024) may limit the generalizability of the results in the training and testing phases of ML models and the performance evaluations in different production scenarios [33]. In contrast, the dataset of 500 samples used in this study allows the models to explore a richer parameter space during training. This enables stronger internal consistency and more stable performance evaluation within the experimental dataset. The materials used in the studies mainly focus on single-type filaments such as PLA or ABS, and comparisons between multiple materials are relatively limited in scope. While studies by Özkül et al. and Kartal et al. have shown high accuracy in specific material groups [41,55], the broad spectrum encompassing 10 different materials such as PLA+, PETG, TPU, rPET, and PP addressed in this study provides broader experimental coverage within a heterogeneous material set. Furthermore, while many studies in the literature focus on only a few output parameters, such as tensile strength or surface roughness, the simultaneous prediction of five mechanical and physical properties in this research makes it a multi-output modeling framework within a single experimental dataset.

Finally, the HMQ-ES-Stack-GBR hybrid model developed achieved an R^2^ value of 0.99, which indicates predictive performance under the defined experimental dataset when compared with baseline ML models such as XGBoost, Random Forest, and ANN trained under the same conditions.

The HMQ-ES-Stack-GBR framework achieved prediction accuracy of R^2^ = 0.99 for the elongation at break output. However, this fit may also indicate a potential risk of overfitting under controlled experimental conditions. Specifically, averaging three physical replicates per parameter combination may reduce output variance and lead to an optimistic estimation of predictive performance by reducing measurement variance. Therefore, the obtained results should be interpreted as an upper-bound performance estimate under laboratory conditions; they should not be interpreted as a claim of absolute generalizability across different production conditions on an industrial scale.

It is important to emphasize that the contribution of the present study is primarily methodological rather than algorithmic. The individual learning algorithms used in the framework, namely XGBoost, Random Forest, and Gradient Boosting Regressor, are well-established methods in the machine learning literature. The novelty of the proposed HMQ-ES-Stack-GBR framework arises from its integration into a unified architecture specifically designed for multi-material and multi-output quality prediction in FDM-based additive manufacturing. In contrast to many previous studies that focus on a single material type, a limited number of quality indicators, or relatively small experimental datasets, the proposed framework combines heterogeneous material groups, output-specific data management, hierarchical stacking, and system-induced variability within a single predictive workflow. Therefore, the primary contribution of this work lies in the design and validation of an application-oriented hybrid ensemble framework capable of representing the complexity of heterogeneous additive manufacturing data under controlled experimental conditions.

Feature significance analysis results show that material type alone explains approximately 55% of the total variance. This finding confirms that, in FDM processes, the chemical structure, crystallinity level, and rheological properties of the polymer have a greater effect on quality outputs than process parameters. This statistical result highlights the dominant role of material-driven variability in the dataset, and because structural properties dominate, the framework is inherently inclined to reflect these inherent material differences rather than artificially overcoming them. Consequently, the model exhibits a material-aware diagnostic behavior. In future studies, a key direction is to further disentangle the contributions of material effects and process parameters by including additional descriptors of microstructure and thermal history in the model.

To transition from a predictive system that reflects and diagnoses quality deviations to an active system that addresses them in real-time, future researchers are advised to focus on autonomous process control systems. By integrating the HMQ-ES-Stack-GBR architecture with real-time sensor fusion (acoustic emission, thermal imaging, and vibration data) and inverse-optimization algorithms, future frameworks could aim to adapt printing parameters on the fly. Furthermore, extending the proposed multi-material library to a wider range of raw materials, such as metal-reinforced filaments or advanced ceramic composites, with DL-based image processing techniques, is considered a strategic research direction towards developing more robust Digital Twin frameworks for smart factories.

A computational performance analysis was conducted to evaluate the practical feasibility of the proposed framework. The HMQ-ES-Stack-GBR model required approximately 4–5 s for training per target output, while inference times remained below 0.1 ms per sample. In addition, the final model size ranged between 8.3 MB and 12.1 MB. These results indicate that the framework imposes relatively low computational overhead and can generate predictions almost instantaneously after training, supporting its potential use in pre-process decision-support systems and future real-time manufacturing applications.

### 4.1. Validation Scope and Study Boundaries

This study is conducted within a controlled experimental and data-driven framework, and the validation scope is intentionally restricted to the dataset generated under defined laboratory conditions. The performance evaluation of the proposed HMQ-ES-Stack-GBR model is based on an internal hold-out validation strategy (80/20 split) applied to the same aggregated dataset. Accordingly, the reported results represent internal predictive performance, rather than external generalization across independent datasets or real-world industrial environments.

A primary limitation of this study is the absence of validation using an external independent dataset. All model development, training, and testing procedures were performed within a single experimentally generated dataset. This may introduce implicit dataset-specific learning biases, which can affect the generalizability of the model beyond the current experimental context.

In addition, the dataset exhibits class imbalance across material groups, where certain polymers (e.g., PLA+) are substantially overrepresented compared with lower-frequency materials such as PP and rPET. Such imbalance may influence the learning behavior of the model and lead to a bias toward dominant material classes during the training process. Because the present study is formulated as a regression problem rather than a classification task, conventional imbalance mitigation techniques such as class weighting, oversampling, and resampling were not applied during model development. Material type was explicitly incorporated as an input feature, allowing the framework to learn material-specific relationships directly from the available data. Nevertheless, the heterogeneous distribution of material classes may still contribute to stronger representation of dominant materials within the learned feature space. Therefore, future studies should investigate more balanced sampling strategies and external validation datasets to further assess the robustness of the framework across underrepresented material groups.

Similarly, the distribution of samples across printer systems is not uniform. Although both open- and closed-case 3D printing systems are included in the dataset, closed-system samples constitute the majority of the data. Therefore, comparisons between printer types should be interpreted as system-induced variability within a controlled experimental dataset, rather than evidence of a fully balanced experimental design for assessing hardware independence.

Furthermore, although the proposed model integrates data from both printer systems to capture process variability, full hardware independence has not been experimentally validated. The observed differences between printer types are primarily attributable to controlled experimental conditions and environmental sensitivity, rather than generalized industrial-level robustness across heterogeneous hardware systems.

Despite these limitations, the study maintains methodological consistency through standardized preprocessing procedures, uniform measurement protocols, and rigorous statistical data sanitization using the IQR method. These measures ensure that the comparative evaluation of machine learning models is conducted under a fair and consistent experimental framework.

Overall, the proposed framework should be interpreted as a diagnostic predictive model validated under controlled laboratory conditions, rather than a fully generalized industrial decision-support system. Future research should focus on external dataset validation, balanced material sampling strategies, and multi-site industrial experiments to further enhance model generalizability and robustness. Although several target variables yielded R^2^ values approaching 0.99, these results should be interpreted within the context of the current experimental design. The combination of controlled laboratory conditions, replicate averaging, and internal validation may contribute to optimistic performance estimates. Nevertheless, the use of 5-fold cross-validation, OOF prediction generation, and an independent hold-out test set was intended to reduce overfitting risk and information leakage. Future studies should incorporate uncertainty quantification, learning-curve analysis, robustness testing under distribution shifts, and validation using independent external datasets to further assess model reliability and generalizability.

#### Effect of Replicate Averaging on Model Performance

To further evaluate the influence of replicate averaging on model performance, an additional comparison was conducted using both the averaged dataset (500 parameter combinations) and the raw replicate dataset containing all individual measurements. As shown in Table 8, the predictive performance of the HMQ-ES-Stack-GBR framework remained consistently high across both data structures. While replicate averaging provided modest improvements for certain outputs, particularly hardness and strain at break, the model maintained strong predictive capability when trained and evaluated using the raw replicate measurements. For example, the R^2^ value for peak stress prediction decreased only from 0.999 to 0.978, while the modulus prediction performance remained nearly unchanged (R^2^ = 0.994 vs. 0.986). These findings indicate that the reported predictive performance is not solely attributable to the averaging procedure and that the proposed framework is capable of capturing the underlying relationships present in both averaged and raw experimental datasets.

## 5. Conclusions

This study proposed the HMQ-ES-Stack-GBR framework for predicting mechanical and physical quality outputs in multi-material FDM 3D printing processes. The framework integrates XGBoost, Random Forest, and Gradient Boosting Regressor within a hierarchical stacking architecture and was evaluated using a dataset comprising 500 validated parameter combinations generated from 10 different filament types.

The main findings of the study can be summarized as follows:

1. The proposed framework achieved strong predictive performance for all investigated quality outputs within the studied dataset. R^2^ values reached 0.999 for peak stress and strain at break, 0.994 for modulus, 0.991 for hardness, and 0.936 for Surface Roughness.
2. Comparative analyses demonstrated that the HMQ-ES-Stack-GBR framework outperformed the baseline models evaluated in this study, including XGBoost, Random Forest, Gradient Boosting Regressor, SVR, KNN, and conventional linear regression approaches.
3. Feature importance analysis revealed that material type was the dominant factor affecting quality outputs, accounting for approximately 55% of the total feature contribution. This finding highlights the importance of material-dependent variability in FDM processes.
4. The inclusion of both open- and closed-frame printer systems enabled the framework to capture system-induced variability within the experimental dataset. For the investigated PLA, PLA+, and PETG materials, higher prediction errors were observed in open-frame printing conditions than in closed-frame systems.
5. To bridge the gap between predictive accuracy and practical shop-floor applications, a multi-objective optimization framework driven by three meta-heuristic algorithms (NSGA-II, PSO, and GWO) was successfully coupled with the predictive system. This optimization pipeline yielded a robust, material-specific process design guideline that defines the precise combinations of infill patterns, layer thickness, printing speeds, and infill densities required to achieve targeted multi-output mechanical and physical properties across the 10 investigated filament classes.

Although the results demonstrate the potential of hybrid ensemble learning for multi-material quality prediction in FDM manufacturing, several limitations remain. The study relied on an internally validated dataset and did not include external validation using independent datasets or unseen printer configurations. Future studies should therefore focus on external validation, balanced material sampling strategies, incorporation of nozzle and bed temperature as independent process variables, and integration of real-time sensor data such as thermal imaging, vibration, acoustic emission, and power consumption measurements. These developments may contribute to more robust data-driven decision-support systems for intelligent manufacturing environments. In addition, future studies will incorporate XAI techniques such as SHAP analysis, feature attribution methods, and partial dependence plots to improve the interpretability of model predictions and better understand material-specific and parameter-dependent prediction behavior. Although the printer system was included as an input feature to capture equipment-related variability, the transferability of the developed model to completely unseen printer platforms requires further validation using independent datasets. Although the printer system was included as an input feature to capture equipment-related variability, the transferability of the developed model to completely unseen printer platforms requires further validation using independent datasets. The lower performance of SVR does not necessarily indicate insufficient optimization; rather, it reflects the limitation of kernel-based approaches in capturing complex interactions among multiple material categories, printer systems, and process parameters. Ensemble tree-based models provided superior flexibility by partitioning the feature space into nonlinear decision regions.

## Figures and Tables

**Table 1:** Comprehensive operational standards, production parameters, and calibration-validated protocols.

Process/ Metric	Standard/ Reference	Equipment/ Calibration	Operational Details & Parameter Levels	Error and Noise Control (Generalization Strategy)
Specimen Geometry	ISO 527-2 [66] Type 4 (Equiv. to ASTM D638)	FDM 3D Printers	10 material types and 500 parameter combinations, resulting in 1500 specimens.	Three independent replicates per configuration were used to reduce experimental variance and improve statistical reliability within the studied dataset.
Printer Systems	Hardware-aware for specific architectures	Creality Ender-3 Pro (Open) & Creality K1 (Closed)	Comparative evaluation of open and closed systems to assess thermal and hardware-induced variability.	Variability across printer types was incorporated into the dataset, enabling the model to learn system-related deviations specifically for the compared systems.
Production Parameters	Systematic Experimental Design	4 Independent Variables	Layer Thickness: 0.1, 0.2, 0.3 mm; Infill: 20, 50, 80%; Speed: 30, 50, 70 mm/s; Pattern: Zigzag, Grid, Triangles.	Nonlinear parameter interactions were modeled using an ensemble learning framework to capture system-induced variability.
Tensile Testing	ISO 527-2 [66]/ASTM D638	MTS Criterion Series Model 45 Universal Testing Machine	Conducted in an accredited laboratory; peak stress, modulus, and strain at break were measured. ISO 527-2 Type 4 specimens; Test speed: 50 mm/min for TPU, 5 mm/min for all other rigid polymers.	All equipment was calibrated in accordance with accredited standards prior to testing to ensure measurement consistency.
Hardness	ISO 868 [67]/ASTM D2240	PCE Instruments Shore D Digital Durometer (Verified with calibration blocks)	Mean of five different measurements taken from different surface locations.	Multi-point sampling was applied to reduce local measurement variability.
Surface Roughness	ISO 21920-2:2021 [68]	TR200 (Calibrated with reference specimen)	Average of three independent measurements per specimen.	Outlier filtering and consistency checks were applied to remove non-representative values.
HMQ-ES-Stack-GBR Algorithm	Hybrid Ensemble Architecture	Two-Level Stacking Framework	Integration of XGBoost, Random Forest, and Gradient Boosting Regressor trained on a multi-material, multi-parameter dataset.	Out-of-Fold (OOF) predictions were used for meta-learner training, reducing overfitting and limiting information leakage under internal validation.

**Table 2:** Hyperparameters used and training configurations.

Model	Hyperparameter	Used Value
Ridge	alpha	1.0
ElasticNet	alpha	0.01
ElasticNet	l1_ratio	0.5
SVR	C	50
SVR	epsilon	0.1
KNN	n_neighbors	7
Decision Tree	max_depth	10
Random Forest	n_estimators	300
Random Forest	random_state	42
GBR	n_estimators	300
XGBoost	n_estimators	500
XGBoost	max_depth	5
XGBoost	learning_rate	0.05
XGBoost	subsample	0.9
XGBoost	colsample_bytree	0.9
XGBoost	objective	reg:squarederror
XGBoost	random_state	42
HMQ-ES-Stack-GBR	Base learners	XGBoost + Random Forest + GBR
HMQ-ES-Stack-GBR	Meta learner	GradientBoostingRegressor
HMQ-ES-Stack-GBR	Meta learner n_estimators	200
HMQ-ES-Stack-GBR	CV strategy	5-fold KFold
HMQ-ES-Stack-GBR	shuffle	True
HMQ-ES-Stack-GBR	random_state	42

**Table 3:** Repeatability analysis of replicate measurements using coefficient of variation.

Quality Output	Mean CV (%)	Median CV (%)
Peak Stress	2.89	2.08
Strain at Break	3.00	3.41
Modulus	2.83	1.65
Hardness	2.95	1.76
Surface Roughness	11.22	8.77

**Table 4:** Comparison of the performance of the HMQ-ES-Stack-GBR model according to its target parameters.

Target	$R^{2}$	MAE	RMSE
Strain at Break (mm/mm)	0.999	0.0006	0.0009
Peak Stress (kPa)	0.999	194.5932	256.1791
Modulus (MPa)	0.994	57.9000	76.4403
Hardness	0.991	1.0843	1.4143
Roughness (um)	0.936	0.4058	0.5290

**Table 5:** Statistical significance analysis of error reduction between baseline models and the proposed HMQ-ES-Stack-GBR using paired *t*-test and Wilcoxon signed-rank test.

Target	Compared Model	Mean Error (Base Model)	Mean Error (HMQ-ES-Stack-GBR)	Paired *t*-Test *p*-Value	Wilcoxon *p*-Value
Roughness	XGBoost	0.77	0.399	1.07 × 10^−35^	8.04 × 10^−49^
	Random Forest	0.781	0.399	1.66 × 10^−38^	6.90 × 10^−52^
	GBR	0.818	0.399	1.28 × 10^−38^	1.73 × 10^−51^
Hardness	XGBoost	2.373	1.087	3.41 × 10^−28^	3.54 × 10^−39^
	Random Forest	2.341	1.087	1.62 × 10^−24^	1.08 × 10^−38^
	GBR	2.491	1.087	2.08 × 10^−29^	1.17 × 10^−43^
Peak Stress (kPa)	XGBoost	461.817	194.489	6.70 × 10^−42^	5.08 × 10^−50^
	Random Forest	608.371	194.489	1.75 × 10^−59^	4.45 × 10^−60^
	GBR	458.992	194.489	1.39 × 10^−45^	2.80 × 10^−51^
Strain at Break (mm/mm)	XGBoost	0.036	0.0006	6.30 × 10^−8^	1.21 × 10^−38^
	Random Forest	0.022	0.0006	1.79 × 10^−5^	9.01 × 10^−20^
	GBR	0.025	0.0006	7.85 × 10^−6^	4.06 × 10^−22^
Modulus (MPa)	XGBoost	126.667	58.369	9.36 × 10^−28^	2.31 × 10^−42^
	Random Forest	138.387	58.369	1.27 × 10^−25^	6.74 × 10^−41^
	GBR	134.065	58.369	1.67 × 10^−34^	5.52 × 10^−51^

**Table 6:** Material-specific optimized process parameter combinations and multi-output quality performance benchmarks.

Material Type	Optimization Algorithm	Infill Pattern	Layer Thickness (mm)	Printing Speed (mm/s)	Infill Density (%)	Surface Roughness (Ra, µm)	Indentation Hardness (Shore D)	Peak Stress (kPa)	Strain at Break (mm/mm)	Elastic Modulus (MPa)	Holistic Optimization Score
TPU	NSGA-II	Zigzag	0.30	30.19	79.23	4.11	34.67	32,855.90	4.2386	297.57	490.25
PLA+	PSO	Quarter Cubic	0.22	51.77	80.00	4.58	78.96	50,570.28	0.0138	4280.94	169.14
PLA-transparent	GWO	Zigzag	0.23	55.45	80.00	5.31	81.50	48,983.38	0.0138	4210.60	168.66
PLA (standard)	PSO	Zigzag	0.29	43.15	80.00	5.77	80.22	44,438.96	0.0121	4169.40	161.79
PLA-phosphorus	GWO	Zigzag	0.23	36.48	80.00	5.24	81.74	46,061.43	0.0131	3779.74	161.68
PLA-CF	PSO	Triangles	0.30	60.12	66.66	4.87	77.75	31,934.11	0.0085	4396.80	149.63
ABS	GWO	Zigzag	0.26	53.79	20.00	7.23	77.92	39,089.77	0.0155	3392.38	145.25
PETG	PSO	Zigzag	0.30	50.37	80.00	5.17	73.68	44,062.41	0.0185	2675.99	141.18
rPET	NSGA-II	Triangles	0.25	69.58	20.80	5.36	55.75	33,004.79	0.0176	2732.74	112.49
PP	GWO	Grid	0.25	61.42	20.00	3.09	35.69	13,043.73	0.0140	1929.23	66.34
PLA+ (Open)	NSGA-II	Triangles	0.21	57.42	78.28	3.88	83.01	43,868.00	0.0119	4097.00	165.17
PLA (Open)	NSGA-II	Triangles	0.21	63.66	78.15	4.02	81.74	42,809.00	0.0125	4007.00	161.84
PETG (Open)	PSO	Zigzag	0.25	44.60	22.04	4.40	76.57	40,304.00	0.0183	3426.00	148.56

**Table 7:** Comparative analysis of the current study with similar studies in the literature.

Study	Material(s)	Number of Samples	Framework Type & Best Model	Outlier/Noise Handling Protocol	Level-1 Meta-Learner Type	Prediction Pipeline Strategy	Target Outputs & Performance Metrics
[33]	PLA	27	ANN	None/Not Specified	None (No Stacking Architecture)	Infill density Printing speed Layer thickness	Surface roughness (Percentage error = 0.71) Tensile strength (Percentage error = 1.77)
[41]	ABS	27	KSTAR MLP	None/Not Specified	None	Independent Target-Specific Prediction using Best-Per-Output Model Selection	Hardness (KSTAR R^2^ = 0.99) Surface roughness (KSTAR (R^2^ = 0.92) Tensile strength (MLP R^2^ = 0.99) Bending strength (MLP R^2^ = 0.99)
[44]	PLA+	42	RF and J48	Standard Outlier Filtering	None	Layer height Print speed Nozzle temperature	Surface roughness (R^2^ ≈ 0.93–0.95)
[55]	PLA/With added walnut shells	18	RF	10-fold cross-validation	None	Global Joint Target (Simultaneous optimization of manufacturing parameters)	Tensile strength (R^2^ = 0.92) Modulus of elasticity (R^2^ = 0.92)
[72]	PLA	68	XGBoost paired with SHAP Analysis	Standard Dataset Splitting (80/20 Train/Test)	None	Global Joint Target (Simultaneous nozzle temperature, print speed, and layer height mapping)	Tensile strength (R^2^ = 0.94)
[73]	PLA	27	XGBoost	Standard Validation Approach	None	Global Joint Target (Focus on traditional layer thickness, infill density, and orientation variables)	Tensile strength (R^2^ = 0.97)
[74]	PLA	31	Nonlinear Regression	Desirability Approach	None	Printing speed, layer thickness, extrusion temperature, and infill percentage	Tensile strength (Percentage error = %2.977) Impact resistance (Percentage error = %6.532) Bending strength (Percentage error = %3.474)
[75]	ABS PLA PLA + CF	20	Nonlinear Regression	Desirability Approach	None	Triangular infill patterns across layer thickness, infill density, and speed)	Tensile strength (Percentage error = %8.98) Modulus of elasticity (Percentage error = %4.39)
This study	PLA+, PLA, PETG, ABS, TPU, PLA-CF, PLA-PHOS, PLA-CLR, rPET, PP	500	HMQ-ES-Stack-GBR	Removal of catastrophic failures and sensor anomalies; IQR-based outlier filtering (Q1–1.5×IQR or above Q3+1.5×IQR); replicate averaging for smart matrix construction.	GBR trained on 5-fold cross-validation-based OOF predictions.	Output-specific independent prediction pipeline with data subclustering and localized error optimization to prevent cross-property noise.	Peak Stress (R^2^ = 0.99) Strain at Break (R^2^ = 0.99) Elastic Modulus (R^2^ = 0.99) Hardness (R^2^ = 0.99) Surface Roughness (R^2^ = 0.93)

**Table 8:** Comparison of HMQ-ES-Stack-GBR performance obtained using averaged and raw replicate datasets.

Target	Averaged R^2^	Raw R^2^
Surface Roughness	0.936	0.947
Hardness	0.991	0.946
Peak Stress	0.999	0.978
Strain at Break	0.999	0.960
Modulus	0.994	0.986

## Data Availability

The original data presented in the study are openly available in Mendeley Data at doi: 10.17632/zd6td6svd6.1.

## Abbreviations

The following abbreviations are used in this manuscript:

3D	Three-Dimensional
AM	Additive Manufacturing
FDM	Fused Deposition Modeling
ISO	International Organization for Standardization
IQR	Interquartile Range
ML	Machine Learning
DL	Deep Learning
ANN	Artificial Neural Network
MLP	Multilayer Perceptron
KNN	K-Nearest Neighbors
SVR	Support Vector Regression
RF	Random Forest
GBR	Gradient Boosting Regressor
XGBoost	Extreme Gradient Boosting
PLA	Polylactic Acid
ABS	Acrylonitrile Butadiene Styrene
PETG	Polyethylene Terephthalate Glycol
TPU	Thermoplastic Polyurethane
PP	Polypropylene
PLA-CF	Carbon Fiber Reinforced Polylactic Acid
rPET	Recycled Polyethylene Terephthalate
HMQ-ES-Stack-GBR	Hybrid Multi-Material Quality Ensemble System—Stacking Gradient Boosting Regressor
NSGA-II	Non-dominated Sorting Genetic Algorithm II
OOF	Out-of-Fold
MPE	Mean Percentage Error
RMSE	Root Mean Square Error
MAE	Mean Absolute Error
R^2^	Coefficient of Determination
Ra	Arithmetic Average Surface Roughness

## References

[1] Aktepe E., Ergün U., Kocer S., Dundar O. (2015). A systematic analysis of 3D printing research in doctoral and specialization theses in Turkey. Next Generation Engineering: Smart Solutions and Applications.

[2] Ngo T.D., Kashani A., Imbalzano G., Nguyen K.T.Q., Hui D. (2018). Additive Manufacturing (3D Printing): A Review of Materials, Methods, Applications and Challenges. Compos. B Eng..

[3] Zohdi N., Yang R.C. (2021). Material Anisotropy in Additively Manufactured Polymers and Polymer Composites: A Review. Polymers.

[4] Bouhamed A., Dammak M., Hagui H., Jrad H. (2025). Multiscale Mechanical Characterization of 3D-Printed PLA Composites with Carbon Fiber Reinforcement: Effect of Raster Angle and Layer Thickness. Int. J. Adv. Manuf. Technol..

[5] Garg S., Sardar A., Srivastava R., Verma S., Madan A.K. (2023). Variation in Tensile Strength of 3D Printed PLA Parts by Varying Infill Density and Infill Pattern. Saudi J. Eng. Technol..

[6] Lorkowski L., Wybrzak K., Brancewicz-Steinmetz E., Świniarski J., Sawicki J. (2025). Influence of Print Speed on the Mechanical Performance of 3D-Printed Bio-Polymer Polylactic Acid. Materials.

[7] Lokesh N., Praveena B.A., Sudheer Reddy J., Vasu V.K., Vijaykumar S. (2022). Evaluation on Effect of Printing Process Parameter through Taguchi Approach on Mechanical Properties of 3D Printed PLA Specimens Using FDM at Constant Printing Temperature. the Materials Today: Proceedings.

[8] Aktepe E., Aktepe Ş. (2024). Comparison of Dimensional Accuracy and Shrinkage Performance of PLA and Recycled PET Filaments in 3D FDM Printing. Int. J. 3D Print. Technol. Digit. Ind..

[9] Aktepe E., Ergün U. (2025). Machine Learning Approaches for FDM-Based 3D Printing: A Literature Review. Appl. Sci..

[10] Alli Y.A., Anuar H., Manshor M.R., Okafor C.E., Kamarulzaman A.F., Akçakale N., Mohd Nazeri F.N., Bodaghi M., Suhr J., Mohd Nasir N.A. (2024). Optimization of 4D/3D Printing via Machine Learning: A Systematic Review. Hybrid Adv..

[11] Goh G.D., Sing S.L., Yeong W.Y. (2021). A Review on Machine Learning in 3D Printing: Applications, Potential, and Challenges. Artif. Intell. Rev..

[12] Rojek I., Mikołajewski D., Kempiński M., Galas K., Piszcz A. (2025). Emerging Applications of Machine Learning in 3D Printing. Appl. Sci..

[13] Ukwaththa J., Herath S., Meddage D.P.P. (2024). A Review of Machine Learning (ML) and Explainable Artificial Intelligence (XAI) Methods in Additive Manufacturing (3D Printing). Mater. Today Commun..

[14] Zhang X., Chu D., Zhao X., Gao C., Lu L., He Y., Bai W. (2024). Machine Learning-Driven 3D Printing: A Review. Appl. Mater. Today.

[15] Liu P., Chang B., Xu C., Zhang W., Yang T., Wu T. (2025). Optimizing 3D Printed Continuous CF/PEEK Composites: A Machine Learning Approach to Strength Prediction. J. Reinf. Plast. Compos..

[16] Zhang J., Wang P., Gao R.X. (2019). Deep Learning-Based Tensile Strength Prediction in Fused Deposition Modeling. Comput. Ind..

[17] Nikzad M.H., Heidari-Rarani M., Rasti R., Sareh P. (2025). Machine Learning-Driven Prediction of Tensile Strength in 3D-Printed PLA Parts. Expert Syst. Appl..

[18] Da Silva M.A., Amaro Junior B., Medeiros R.R.B., Pinheiro P.R. (2022). A Neuroevolutionary Model to Estimate the Tensile Strength of Manufactured Parts Made by 3D Printing. Algorithms.

[19] Pazhamannil R.V., Govindan P., Sooraj P. (2019). Prediction of the Tensile Strength of Polylactic Acid Fused Deposition Models Using Artificial Neural Network Technique. Proceedings of the Materials Today: Proceedings.

[20] Aktepe E., Koca Y.B. (2025). Optimization of 3D Printing Parameters Using Machine Learning Techniques. Proceedings of the EAI/Springer Innovations in Communication and Computing.

[21] Ridlo A.H., Kusmono, Muflikhun M.A., Putra R.A., Thongking W., Wiranata A. (2025). Optimization of Stretchable Fused Deposition Modeling Filament From Polypropylene-Based Elastomer/Thermoplastic Elastomer Blends Using a Machine Learning Approach. J. Appl. Polym. Sci..

[22] Wang Z., Yang Y., Suo S., Guo J., Rao W.F. (2024). Predicting 4D Hardness Property from 3D Datasets for Performance-Tunable Material Extrusion Additive Manufacturing. Mater. Today Commun..

[23] Veeman D., Vellaisamy M., Ponnusamy P.C., Subathra D.P., Katiyar J.K. (2025). Impact of Process Parameters on Hardness of Melt Fabricated PA6 Carbon Fiber-Reinforced Composites and Prediction of Properties Using Machine Learning. J. Mater. Eng. Perform..

[24] Zeng Y.S., Hsueh M.H., Lai C.J., Hsiao T.C., Pan C.Y., Huang W.C., Chang C.H., Wang S.H. (2022). An Investigation on the Hardness of Polylactic Acid Parts Fabricated via Fused Deposition Modeling. Polymers.

[25] Reddy B.V.S., Shaik A.M., Sastry C.C., Krishnaiah J., Patil S., Nikhare C.P. (2025). Performance Evaluation of Machine Learning Techniques in Surface Roughness Prediction for 3D Printed Micro-Lattice Structures. J. Manuf. Process..

[26] Sharma A., Bharti P.S. (2025). Optimization of Resin Printing Parameters for Improved Surface Roughness Using Metaheuristic Algorithms: A Multifaceted Approach. J. Mater. Eng. Perform..

[27] Xie S., He Z., Wang C., Liu C., Ke X. (2023). A Generic Evolutionary Ensemble Learning Framework for Surface Roughness Prediction in Manufacturing. Int. J. Comput. Integr. Manuf..

[28] Kim M.K., Lee I.H., Kim H.C. (2018). Effect of Fabrication Parameters on Surface Roughness of FDM Parts. Int. J. Precis. Eng. Manuf..

[29] Abdulshahed A.M., Wafa F. (2025). Surface Roughness Prediction in Additive Manufacturing: Presenting the Power of Neural Networks Compared to Linear Regression. J. Adv. Manuf. Syst..

[30] Kharate N., Anerao P., Kulkarni A., Abdullah M. (2024). Explainable AI Techniques for Comprehensive Analysis of the Relationship between Process Parameters and Material Properties in FDM-Based 3D-Printed Biocomposites. J. Manuf. Mater. Process..

[31] Khusheef A.S., Shahbazi M., Hashemi R. (2024). Predicting 3D Printed Plastic Part Properties: A Deep Learning Approach with Thermographic and Vibration Data Fusion. Expert Syst. Appl..

[32] Marappan K., Jenarthanan M.P., Begum K.G., Moorthy V. (2024). Prediction of Effective Parameters for 3D Printing of Poly Lactic Acid-Carbon Fibre Composites Using Intelligent Frameworks Based on Mechanical Response. Pigment Resin Technol..

[33] Selvan S.P., Raja D.E., Muthukumar V., Sonar T. (2024). Optimization of Process Parameters and Predicting Surface Finish of PLA in Additive Manufacturing—A Neural Network Approach. Int. J. Interact. Des. Manuf..

[34] Wolpert D.H. (1992). Stacked Generalization. Neural Netw..

[35] Breiman L. (1996). Stacked Regressions. Mach. Learn..

[36] Friedman J.H. (2001). Greedy Function Approximation: A Gradient Boosting Machine. Ann. Stat..

[37] Mulugundam S.S., Gugulothu S.K., Varshith M. (2025). A Machine Learning Approach to Refining Surface Quality and Material Durability in Additive Manufacturing. Prog. Addit. Manuf..

[38] Ogunsanya M.A., Parupelli S.K., Isichei J.C., Desai S. (2025). Predictive Quality Assurance of Material Extrusion Process Using Artificial Neural Networks and Ensemble Models. Int. J. Adv. Manuf. Technol..

[39] Nguyen P.Q.K., Zhang Y.S., Zhang Z., Yang R.C. (2025). Multi-Objective Optimisation of Fused Filament Fabrication of Acrylonitrile Butadiene Styrene for Enhancing Mechanical Performance and Build Time. Int. J. Adv. Manuf. Technol..

[40] Panico A., Corvi A., Collini L., Sciancalepore C. (2025). Multi Objective Optimization of FDM 3D Printing Parameters Set via Design of Experiments and Machine Learning Algorithms. Sci. Rep..

[41] Özkül M., Kuncan F., Ulkir O. (2025). Predicting Mechanical Properties of FDM-Produced Parts Using Machine Learning Approaches. J. Appl. Polym. Sci..

[42] Nagarjun J., Saravanakumar N., Thirumalai Kumaran S., Anto Dilip A., Balasuadhakar A. (2025). Empirical Study and Machine Learning Prediction of Tensile Strength in 3D Printed Eco-Friendly Polylactic Acid. Prog. Rubber Plast. Recycl. Technol..

[43] Saylık A., Kösedağ E., Etem T. (2025). Copula-Based Data Augmentation and Machine Learning for Predicting Tensile Strength of 3D-Printed PLA Under Anisotropic Conditions. J. Appl. Polym. Sci..

[44] Soundararajan R., Sathishkumar A., Aathil S.A., Chandran N.G. (2025). Evaluating Machine Learning Methods for Predicting Surface Roughness of FDM Printed Parts Using PLA plus Material. Int. J. Interact. Des. Manuf..

[45] Sharma V., Singh J., Sharma R.C., Singh A. (2025). Machine Learning-Based Prediction and Optimization of Surface Roughness in FDM-Printed PLA Using ANN and Box-Behnken Design. Mater. Sci. Technol..

[46] Tzotzis A., Nedelcu D., Mazurchevici S.N., Kyratsis P. (2025). Investigating the Machining Behavior of the Additively Manufactured Polymer-Based Composite Using Adaptive Neuro-Fuzzy Learning. Appl. Sci..

[47] Islahudin N., Nugroho D.S., Wijaya D.K., Amalia, Suprijono H., Ginta T.L., Azka M., Rahadian H. (2025). Machine Learning-Driven Optimization for Surface Roughness Prediction of Vertical Orientation Measurements on 3D Printed Components. Clean. Eng. Technol..

[48] Mohammed Raffic N., Ganesh Babu K., Dharani Kumar S., Parrthipan B.K. (2025). Surface Roughness and Printing Time Minimization in 3D Printed Aramid Fiber Reinforced Polyamide Parts through Taguchi-CoCoSo-Machine Learning Techniques. J. Mater. Eng. Perform..

[49] Vishwadarshan, Shetty G., Shetty R., Supriya J.P., Balaji V., Hegde A. (2025). Comprehensive Analysis of Drilling Responses in Additively Manufactured PLA Using a Regression—Based Statistical Learning Approach. Mater. Res. Express.

[50] Sharma A., Saini R.S., Kaushik A., Okshah A., Kuruniyan M.S., Gurumurthy V., Vyas R., Binduhayyim R.I.H., Heboyan A. (2025). Machine Learning Based Approach for Surface Roughness Prediction in Precision Dental Prototyping. Sci. Rep..

[51] Patil S., Sathish T., Nelson M., Ghodhbani R., Tarawneh B., Othman N.A. (2025). Optimization and Prediction of Tensile Strength in 3D-Printed PLA/HAp Composites Using Response Surface Methodology and Integrated Machine Learning Technique. J. Appl. Polym. Sci..

[52] Ozkul M., Kuncan F., Ulkir O. (2025). Predictive Modeling of Additively Manufactured Carbon Fiber-PLA Mechanical Components via ML. Multidiscip. Model. Mater. Struct..

[53] Raffic Noor Mohamed M., Karuppiah G.B., Selvan D.K., Saminathan R., Sharma S., Dwivedi S.P., Kumar S., Abbas M., Kozak D., Lozanovic J. (2025). Exploring the Process—Structure–Property Relationship of Nylon Aramid 3D Printed Composites and Parameter Optimization Using Supervised Machine Learning Techniques. J. Eng. Fibers Fabr..

[54] Shi D., Xu K., Chao Z., Cui P. (2025). Experimental Study on Mechanical Properties of Triaxial Geogrid Reinforced Marine Coral Sand-Clay Mixture Based on 3D Printing Technology. Front. Mar. Sci..

[55] Kartal F. (2025). Mechanical Performance Optimization in FFF 3D Printing Using Taguchi Design and Machine Learning Approach with PLA/Walnut Shell Composites Filaments. J. Vinyl Addit. Technol..

[56] Razzaq M.H., Zaheer M.U., Asghar H., Aktas O.C., Aycan M.F., Mishra Y.K. (2025). Additive Manufacturing for Biomedical Bone Implants: Shaping the Future of Bones. Mater. Sci. Eng. R Rep..

[57] Ziadia A., Habibi M., Kelouwani S. (2025). Digital Twin-Driven Real-Time Optimization of Layer-Specific Surface Roughness in FDM 3D Printing. Prog. Addit. Manuf..

[58] Tran N.H., Phan N.D.M. (2025). Analyzing the Impact of Process Parameters on Surface Roughness and Mechanical Properties in FDM 3D Printing Using Machine Learning. Int. J. Interact. Des. Manuf..

[59] Kumar S., Kumar R. (2025). A Comprehensive Study on Additive Manufacturing Techniques, Machine Learning Integration, and Internet of Things-Driven Sustainability Opportunities. J. Mater. Eng. Perform..

[60] Guirguis D., Tucker C., Beuth J. (2024). Accelerating Process Development for 3D Printing of New Metal Alloys. Nat. Commun..

[61] Nasrin T., Pourali M., Pourkamali-Anaraki F., Peterson A.M. (2023). Active Learning for Prediction of Tensile Properties for Material Extrusion Additive Manufacturing. Sci. Rep..

[62] Aktepe E., Ergün U. (2026). Deep Learning Application for Image-Based Defect Detection in 3D Printing Processes. J. Mater. Mechatron. A.

[63] Saleh S., Guo Y.B., Guo W. (2025). “Grace” Enhanced Counterfactual Explanations for Optimizing Three-Dimensional Printing Parameters Using SHAP and Nearest-Neighbor Constraints With Physics-Based Validation. J. Manuf. Sci. Eng..

[64] Fang Q., Yu J., Liu Y., Shi B. (2026). Machine Learning and SHAP for Predicting Impact Strength of FDM-Printed Short Carbon Fiber-Reinforced Nylon. J. Reinf. Plast. Compos..

[65] Abbili K.K. (2024). Explainable Artificial Intelligence (XAI) and Machine Learning Technique for Prediction of Properties in Additive Manufacturing. J. Adv. Manuf. Syst..

[66] (2012). Plastics—Determination of Tensile Properties—Part 2: Test Conditions for Moulding and Extrusion Plastics.

[67] (2003). 2003: Plastics—Determination of Indentation Hardness by Means of a Durometer (Shore Hardness).

[68] (2021). 2021 Specifications, Geometrical Product. “Surface Texture: Profile—Part 2: Terms, Definitions and Surface Texture Parameters”.

[69] Deb K., Pratap A., Agarwal S., Meyarivan T. (2002). A Fast and Elitist Multiobjective Genetic Algorithm: NSGA-II. IEEE Trans. Evol. Comput..

[70] Kennedy J., Eberhart R.C. A Discrete Binary Version of the Particle Swarm Algorithm. Proceedings of the 1997 IEEE International Conference on Systems, Man, and Cybernetics. Computational Cybernetics and Simulation.

[71] Mirjalili S., Mirjalili S.M., Lewis A. (2014). Grey Wolf Optimizer. Adv. Eng. Softw..

[72] Ege D., Sertturk S., Acarkan B., Ademoglu A. (2023). Machine Learning Models to Predict the Relationship between Printing Parameters and Tensile Strength of 3D Poly (Lactic Acid) Scaffolds for Tissue Engineering Applications. Biomed. Phys. Eng. Express.

[73] Jayasudha M., Elangovan M., Mahdal M., Priyadarshini J. (2022). Accurate Estimation of Tensile Strength of 3D Printed Parts Using Machine Learning Algorithms. Processes.

[74] Jatti V.S., Sapre M.S., Jatti A.V., Khedkar N.K., Jatti V.S. (2022). Mechanical Properties of 3D-Printed Components Using Fused Deposition Modeling: Optimization Using the Desirability Approach and Machine Learning Regressor. Appl. Syst. Innov..

[75] Tandon S., Singh S.K., Kacker R., Gautam S.S., Tamang S.K. (2025). Multi-Response Optimization of 3D Printed Parts with Triangular Patterns Using Nonlinear Machine Learning Regressor Technique. J. Mater. Eng. Perform..

